# *Zanthoxylum bungeanum* Essential Oil Alleviates Obesity and Dysregulated Lipid Metabolism in Diet-Induced Obese Mice: A Multi-Omics Study Linking Gut Microbiota to Fatty Acid Metabolism

**DOI:** 10.3390/nu18182950

**Published:** 2026-09-09

**Authors:** Luchuanyang Sun, Shiqi Zhang, Hongjuan Hu, Bingbing Xu, Shan Qian, Yukun Huang, Xiao Yang, Yan Liang, Xianggui Chen

**Affiliations:** 1 School of Food and Bio-Engineering, Xihua University, Chengdu 610039, China; 15036872111@163.com (S.Z.); 14708220796@163.com (H.H.); bettyxu121@163.com (B.X.); qians@xhu.edu.cn (S.Q.); hyk_diana@163.com (Y.H.); 13076014204@163.com (X.Y.); 2 School of Food and Pharmacy, Zhejiang Ocean University, Zhoushan 316022, China; 18040107762@163.com; 3 Food Microbiology Key Laboratory of Sichuan Province, Chengdu 610039, China; 4 Chongqing Key Laboratory of Speciality Food Co-Built by Sichuan and Chongqing, Chengdu 610039, China

**Keywords:** *Zanthoxylum bungeanum* essential oil, obesity, gut microbiota, fatty acid metabolism, multi-omics, metabolome

## Abstract

**Background:** Obesity is a metabolic disorder characterized by excessive lipid accumulation, disrupted fatty acid homeostasis, and gut microbiota dysbiosis. *Zanthoxylum bungeanum* essential oil (ZBO) is a natural plant volatile oil with diverse bioactivities, but its anti-obesity effects and potential associations remain unclear. **Methods**: HFD-induced obese (DIO) mice were evaluated to determine the effects of ZBO. Physiological parameters, glucose tolerance, and serum biochemical markers were assessed. Potential correlates were explored using a multi-omics approach, including integrated serum untargeted metabolomics, 16S rRNA gene sequencing, and RT-qPCR analysis of key genes involved in lipid metabolism in the liver and white adipose tissue. **Results**: ZBO significantly mitigated HFD-induced body weight gain, visceral adiposity, hepatic steatosis, dyslipidemia, and glucose intolerance. Metabolomics indicated that ZBO administration was associated with alterations in the serum metabolic landscape, particularly in pathways related to fatty acid metabolism and AMPK/PPAR-α signaling. Consistently, ZBO treatment was associated with upregulated mRNA expression of lipid-oxidizing genes (AMPK, PPAR-α, CPT1) and downregulated lipogenic genes (SREBP-1c, ACC, FAS, SCD1, LPL). 16S rRNA sequencing suggested that ZBO was associated with increased gut microbial diversity, decreased the Firmicutes/Bacteroidota ratio, and enriched beneficial taxa (e.g., Ligilactobacillus, Alistipes) while suppressing pro-inflammatory genera. Correlation analysis established robust associations between ZBO-modulated microbes and key fatty acid metabolites, particularly acylcarnitines and AMPK/PPAR-α-related intermediates. **Conclusions**: Collectively, these multi-omics data suggest that ZBO administration is associated with remodeling of the gut microbiota and correlated changes in fatty acid metabolic pathways. These findings provide preliminary preclinical evidence supporting further investigation of ZBO in the context of dietary strategies for alleviating HFD-induced obesity.

## 1. Introduction

Obesity is a metabolic disorder characterized by excessive lipid accumulation and fundamentally arises from an imbalance between energy intake and energy expenditure [1]. It is closely associated with multiple metabolic disturbances, including dysregulated glucose and lipid metabolism and gut microbiota dysbiosis, which may ultimately contribute to the development of severe diseases such as insulin resistance, type 2 diabetes mellitus, non-alcoholic fatty liver disease, and cardiovascular diseases [2,3,4]. A recent survey projected that, by 2050, the global population with overweight or obesity will reach 3.8 billion, accounting for more than half of the world’s adult population at that time [5]. Obesity has therefore become a major threat to global public health. Current strategies for combating obesity include physical exercise, dietary intervention, and anti-obesity pharmacotherapy. However, sustained lifestyle modification remains a long-term challenge for many individuals. In addition, anti-obesity drugs such as sibutramine and orlistat are associated with adverse health effects [6]. Therefore, there is a substantial need to explore natural therapies for the development of alternative, safer, and more effective strategies. In this context, a variety of natural phytochemicals have attracted increasing attention owing to their potential to enhance fatty acid β-oxidation, regulate lipid absorption, and suppress appetite.

Excessive adipose tissue expansion is closely linked to systemic disruption of fatty acid homeostasis [7]. Chronic energy surplus can induce lipid metabolic reprogramming in the liver and adipose tissue, characterized by enhanced fatty acid synthesis, impaired fatty acid oxidation, and aberrant lipid deposition in non-adipose tissues [8]. Sterol regulatory element-binding protein-1c (SREBP-1c) is a key transcription factor regulating de novo fatty acid synthesis and promotes the expression of lipogenic enzymes such as acetyl-CoA carboxylase (ACC), fatty acid synthase (FAS), and stearoyl-CoA desaturase 1 (SCD1). Meanwhile, malonyl-CoA, generated through ACC-mediated catalysis, inhibits the activity of carnitine palmitoyltransferase 1 (CPT1), thereby restricting the entry of long-chain fatty acids into mitochondria for β-oxidation [9]. Increased fatty acid synthesis, together with insufficient fatty acid oxidation, promotes the accumulation of triglycerides and lipotoxic intermediates in tissues such as the liver and skeletal muscle, leading to mitochondrial dysfunction, endoplasmic reticulum stress, chronic inflammation, and impaired insulin signaling [10,11]. Therefore, improving the dynamic balance between fatty acid synthesis and oxidation represents an important strategy for ameliorating obesity-related metabolic disorders. As central signaling nodes in the regulation of energy metabolism and fatty acid oxidation, AMP-activated protein kinase (AMPK) and peroxisome proliferator-activated receptors (PPARs) can alleviate ectopic lipid deposition and lipotoxic injury by inhibiting lipid synthesis, reducing malonyl-CoA levels, and enhancing CPT1-mediated fatty acid transport and β-oxidation [12,13]. These mechanisms provide a theoretical basis for targeting the AMPK/PPAR-α signaling pathways in the intervention of obesity and its metabolic complications.

Disturbances in fatty acid metabolism are not solely attributable to intrinsic metabolic abnormalities in host tissues but are also shaped by the gut microbiota and its metabolites [14]. A high-fat diet can alter gut microbial diversity and community structure, thereby affecting the production and transformation of short-chain fatty acids, bile acid derivatives, choline metabolites, branched-chain amino acids, and lipid-related small molecules [15,16]. These metabolites can regulate intestinal barrier integrity, endotoxin burden, chronic inflammatory responses, and hepatic lipid metabolism through the gut–liver axis [17,18]. Specifically, short-chain fatty acids participate in AMPK-mediated regulation of energy metabolism, bile acids influence lipid absorption and fatty acid oxidation through farnesoid X receptor/takeda G protein-coupled receptor 5 (FXR/TGR5) signaling, and lipopolysaccharide translocation can activate the toll-like receptor 4/nuclear factor kappa-B (TLR4/NF-κB) inflammatory pathway, thereby promoting insulin resistance and lipid deposition [19,20,21]. Therefore, the gut microbiota may serve as an important regulatory node linking a high-fat diet, inflammatory responses, and abnormal fatty acid metabolism.

*Zanthoxylum bungeanum* Maxim., commonly known as Chinese prickly ash, is a traditional Chinese plant resource used for both medicinal and dietary purposes [22]. Its essential oil, *Zanthoxylum bungeanum* essential oil (ZBO), is rich in volatile bioactive constituents, including linalool, limonene, sabinene, myrcene, and pinene, and exhibits multiple biological activities, such as antioxidant, anti-inflammatory, antibacterial, and gastrointestinal regulatory effects [23]. In our previous study, it was demonstrated that ZBO can modulate dextran sulfate sodium-induced ulcerative colitis by modulating the gut microbiota [24]. In addition, other studies have shown that certain volatile phytochemicals can ameliorate high-fat diet-induced metabolic abnormalities by regulating the gut microbiota, inflammatory responses, lipid absorption, and energy metabolism-related pathways [25]. However, whether ZBO can effectively alleviate high-fat diet-induced obesity and its associated metabolic disorders remains to be systematically investigated. Therefore, in the present study, a diet-induced obesity (DIO) mouse model was used to systematically evaluate the effects of ZBO on body weight gain, fat accumulation, impaired glucose tolerance, dyslipidemia, and liver injury. Furthermore, serum untargeted metabolomics, 16S rRNA sequencing of the gut microbiota, and analysis of lipid metabolism-related gene expression in liver and adipose tissues were integrated to explore potential correlations between ZBO administration and obesity-related metabolic disorders. Particular emphasis was placed on investigating whether ZBO administration was associated with changes in fatty acid metabolic homeostasis, obesity phenotypes, and gut microbiota composition, as well as alterations in lipid-related metabolites and genes involved in fatty acid synthesis and oxidation, including AMPK/PPAR-α, SREBP-1c/ACC/FAS, and CPT1-related signaling.

## 2. Materials and Methods

### 2.1. Materials

*Zanthoxylum bungeanum* essential oil was purchased from Shanghai Yuanye Biotechnology Co., Ltd. (Shanghai, China). The chemical composition, molecular formulas, and relative abundances of ZBO, as determined by gas chromatography-mass spectrometry (GC-MS), were summarized in Table S1. Figures S1 and S2 presented the GC-MS chromatogram of ZBO and the chemical structures of the top 10 compounds ranked by relative abundance, respectively. High-fat diet (D12492) was purchased from Research Diets, Inc. (New Brunswick, NJ, USA). TRIpure Reagent was purchased from Beijing Aide Lai Biotechnology Co., Ltd. (Beijing, China). RIPA Lysis Buffer and BCA Protein Assay Kit were purchased from Kangwei Century Biotechnology Co., Ltd. (Beijing, China). The Ultra-Sensitive ELC Chemiluminescence Assay Kit was purchased from Shanghai Biyun Tian Biotechnology Co., Ltd. (Shanghai, China).

### 2.2. Animal Experiment Design

**Figure 1:**
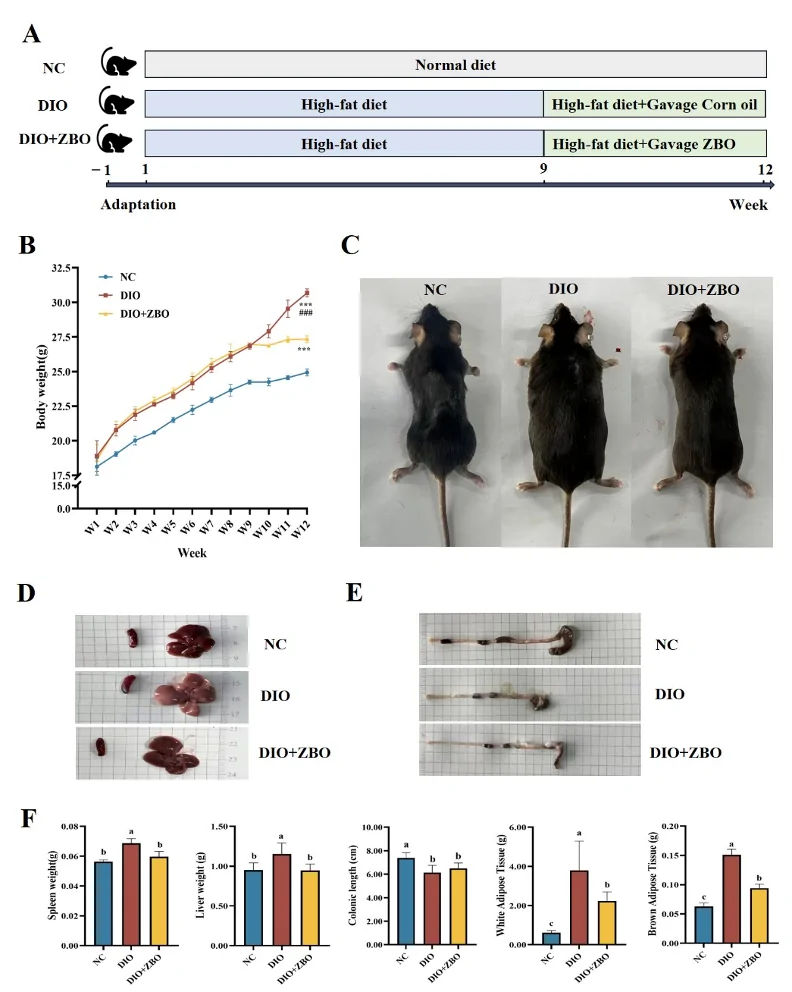
Ameliorative effects of ZBO on body weight and related organ indices in DIO mice. (**A**) Experimental design and grouping scheme. (**B**) Body weight changes in the mice in each group during the experimental period. (**C**) Representative appearances of the mice across groups. (**D**) Representative images of the livers and spleens from each group. (**E**) Representative images of the colons from each group. (**F**) Statistical analysis of organ indices across groups, including spleen weight, liver weight, colon length, white adipose tissue weight, and brown adipose tissue weight. NC, normal maintenance diet; DIO, the high-fat diet group with corn oil; DIO+ZBO, *Zanthoxylum bungeanum* essential oil intervention group. Data are expressed as the mean ± standard deviation. Different lowercase letters indicate significant differences between groups (*p* < 0.05). Compared with the NC group: ***: *p* < 0.001. Compared with the DIO group: ###: *p* < 0.001.

Four-week-old male mice (C57BL/6) were housed in a well-ventilated, pathogen-free, air-conditioned room maintained at 25 ± 1 °C and 55 ± 5% relative humidity. Mice had free access to food and water under a 12 h light/dark cycle. The experimental procedures followed the standards outlined in the Guide for the Care and Use of Laboratory Animals and were approved by the Institutional Animal Care and Use Committee. After one week of adaptive feeding, the experimental mice were divided into three groups (n = 6/group): normal maintenance diet group (NC); the high-fat diet group with corn oil (DIO); *Zanthoxylum bungeanum* essential oil intervention group (DIO+ZBO). During the entire formal experimental period, the NC group was fed SPF-grade maintenance feed daily, while the DIO and DIO+ZBO groups were fed a high-fat diet. From weeks 9 to 12 of the experiment, mice in the DIO+ZBO group received daily oral administration of 40 mg/kg Sichuan pepper essential oil. The entire animal testing process is illustrated in Figure 1A. After 12 weeks of experimentation, mice were fasted for 12 h, whole blood was centrifuged to obtain serum, and tissues such as liver, colon, spleen, white adipose tissue, brown adipose tissue, and cecal contents were collected from mice after cervical dislocation. All samples were stored at −80 °C.

### 2.3. Glucose Tolerance Test (GTT)

The glucose tolerance test (GTT) was conducted at 12 weeks to evaluate glucose tolerance in each group of mice. Mice were fasted for 12 h, and the required glucose solution volume per mouse was calculated based on body weight (2 g/kg). First, measure the fasting blood glucose level of each mouse (0 min). Then, administer a 50% sterile glucose solution via intraperitoneal injection. At 15, 30, 60, and 120 min post-injection, collect blood samples from the tail vein and monitor blood glucose levels using a blood glucose meter. Record the blood glucose concentration of each mouse at each time point.

### 2.4. Serum Biochemical Analysis

Gradually thaw serum stored at −80 °C on ice. Total cholesterol (TC), triglycerides (TG), high-density lipoprotein cholesterol (HDL-C), low-density lipoprotein cholesterol (LDL-C), aspartate aminotransferase (AST), and alanine aminotransferase (ALT) levels were measured using an automated biochemical analyzer (Thermo Scientific Indiko Plus, Waltham, MA, USA).

### 2.5. Hematoxylin and Eosin Staining (H&E)

H&E staining was used to observe pathological changes in liver tissue. Collected liver tissue was fixed in 4% paraformaldehyde solution and dehydrated sequentially with ethanol solutions of varying concentrations. The liver tissue was then paraffin-embedded and sectioned (3–5 μm). After dewaxing with xylene, sections were rehydrated using a graded ethanol series. Intestinal sections were sequentially stained with hematoxylin and eosin solutions. Morphological features of the liver were observed under a microscope, and images were captured.

### 2.6. Real-Time Quantitative Polymerase Chain Reaction (RT-qPCR)

Total RNA was extracted from liver and adipose tissue using the TRIzol method. RNA reverse transcription was performed using the ABScript Neo RT Pre-mix (containing gDNA removal agent, ABclonal Technology, Wuhan, China, RK20433) according to the manufacturer’s instructions to obtain cDNA. Subsequently, RT-qPCR detection was conducted using the 2∈ Universal SYBR Green Rapid qPCR Pre-mix (ABclonal Technology, RK21203). Target gene primers were synthesized by Shanghai Sangon Biotech Co., Ltd. (Shanghai, China) (Table S2). Gapdh was employed as the internal reference gene. Relative gene expression levels were calculated using the 2^−ΔΔCT^ method.

### 2.7. Gut Microbiome Analysis

The collected intestinal contents were analyzed to detect the gut microbiota. The variable region V3–V4 of the 16S rRNA gene was amplified via PCR using primers 338F and 806R. The Vsearch method was employed for primer removal, quality filtering, denoising, assembly, and chimera elimination, ultimately yielding feature sequences (Amplified Sequence Variants—ASVs). Following ASV generation, length distributions were statistically analyzed, and all representative sequences were annotated against databases. Based on the collected data, α-diversity and β-diversity were assessed, and the structure and abundance of the gut microbiota were analyzed. Sequencing depth was assessed by rarefaction curves to ensure sufficient coverage for each sample. To normalize sequencing depth across samples, ASV counts were rarefied to the minimum sequencing depth prior to alpha- and beta-diversity analyses. Taxonomic assignment of representative ASVs was performed using the SILVA database (version 138) with a confidence threshold of 0.7. Beta-diversity was evaluated using both unweighted and weighted UniFrac distances, and statistical differences between groups were tested using PERMANOVA (999 permutations). Differential abundance of taxa at phylum, family, and genus levels was assessed using the Kruskal–Wallis test followed by Dunn’s post hoc test with Benjamini–Hochberg false discovery rate (FDR) correction to control for multiple comparisons (q < 0.05 considered significant). LEfSe analysis was performed with an LDA score threshold of 3.0. Due to the limited sample size (n = 6 per group), all microbiome findings are interpreted as exploratory and hypothesis-generating, pending validation in larger cohorts.

### 2.8. Serum Non-Targeted Metabolomics Analysis

Non-targeted metabolomics analysis of serum was performed using Thermo’s high-resolution LC-MS. First, metabolites were extracted from the serum. Pipette 50 μL of sample into a 2 mL centrifuge tube; Add 200 μL of pre-cooled methanol:acetonitrile (1:1, *v*/*v*) and vortex for 30 s; Freeze in a −20 °C refrigerator for 30 min; Centrifuge at 12,000 rpm and 4 °C for 10 min, take 200 μL of the supernatant and concentrate to drynessunder vacuum; Add 100 μL 50% methanol (containing 5 ppm 2-chlorophenylalanine) to dissolve and vortex for 30 s; Centrifuge at 12,000 rpm and 4 °C for 10 min, filter the supernatant through a 0.22 μm filter membrane, and add the filtrate to the test bottle; Take 10–20 μL of each sample filtrate and mix them into a quality control sample for evaluating instrument stability and data reliability. Then establish chromatographic and mass spectrometry methods for detecting metabolites. An ACQUITY UPLC HSS T3 column (Waters, Milford, MA, USA) (100Å, 1.8 µm, 2.1 mm × 100 mm) was used with a flow rate of 0.4 mL/min, a column temperature of 40 °C, an autosampler temperature of 8 °C, and an injection volume of 2 μL. HESI source, spray voltage 3.5 kV/−3.0 kV, sheath gas 40 arb, auxiliary gas 15 arb, capillary temperature 325 °C, auxiliary gas temperature 300 °C, primary resolution 60,000, scan range 100–1000 *m*/*z*, AGC Target Standard, Max IT 100 ms, the top 4 ions were screened for secondary fragmentation, dynamic exclusion time was 8 s, secondary resolution 15,000, HCD collision energy 30%, AGC Target Standard, Max IT Auto. Finally, the metabolites were subjected to qualitative and quantitative analysis using Compound Discoverer™ 3.3 software (version 3.3.2.31, Thermo, Waltham, MA, USA). Metabolite identification was performed using the Compound Discoverer™ 3.3 software by matching exact mass (*m*/*z*) and retention times against the HMDB database. To ensure data reliability, only metabolites meeting the following criteria were retained for further analysis: mass accuracy deviation < 5 ppm, signal-to-noise ratio > 3, and chromatographic peaks with clear biological origin. All identified metabolites were classified as putative annotations (Confidence Level 2) according to the Metabolomics Standards Initiative (MSI) guidelines. Exogenous interfering peaks that were not biologically plausible were excluded from the final dataset. Prior to statistical analysis, raw metabolomic data were preprocessed using Compound Discoverer™ 3.3, including peak alignment, filtering, and missing value imputation (using half of the minimum positive value per feature). Data were then normalized by total ion intensity and log-transformed to approximate normality. No batch correction was required as all samples were analyzed in a single batch. For multivariate analysis, Pareto scaling was applied. OPLS-DA models were validated using 7-fold cross-validation and 200 permutation tests to assess model overfitting and statistical significance. Metabolite identification confidence was assigned according to the Metabolomics Standards Initiative (MSI) criteria: all reported metabolites are classified as Level 2 (putative annotation based on accurate mass and retention time matching to HMDB), except for a subset of acylcarnitines and fatty acid derivatives that were further confirmed by MS/MS spectral matching (Level 1). For univariate analysis, metabolites with VIP ≥ 1.5 and *p* < 0.05 (Welch’s *t*-test) were considered differentially accumulated. FDR correction (Benjamini–Hochberg) was applied to control the false discovery rate at q < 0.1 for KEGG-enriched metabolites. Given the small group size, the metabolomic findings are presented as exploratory and require independent validation.

### 2.9. Statistical Analysis

All results are expressed as mean ± standard deviation. For data collected at a single time point, one-way analysis of variance (ANOVA) was performed, followed by Duncan’s multiple range test. For data collected at multiple time points across different mouse groups, two-way ANOVA was conducted, with group serving as the between-subjects factor and time point as the within-subjects factor, followed by Duncan’s multiple range test. All statistical analyses were performed using SPSS software 26.0 and Excel software, with graphical representations generated using GraphPad Prism 10 software. For high-dimensional omics data (metabolomics and microbiome), differential metabolites were screened using orthogonal partial least squares discriminant analysis (OPLS-DA) with variable importance in projection (VIP) ≥ 1.5 and *p* < 0.05 as selection criteria. For correlation analyses between microbiota and metabolites, Spearman’s rank correlation coefficients were calculated, and only correlations with |r| > 0.6 and *p* < 0.05 were considered significant. All omics analyses were performed using R software (version 4.2.1) with appropriate packages (e.g., vegan, phyloseq, MetaboAnalystR). To mitigate the risk of type I errors arising from multiple comparisons across numerous endpoints, all intergroup comparisons were conducted within the framework of ANOVA followed by Duncan’s multiple range test, which provides conservative control of the family-wise error rate at the level of each individual endpoint. For multi-omics data, stringent filtering criteria (VIP ≥ 1.5 and *p* < 0.05 for metabolomics; Spearman’s |r| > 0.6 and *p* < 0.05 for correlations) were applied to reduce the number of candidates subjected to multiple testing.

## 3. Results

### 3.1. ZBO Was Associated with Alleviation of Obese Phenotypes in DIO Mice

To investigate the effects of ZBO on established HFD-induced obesity, we established a high-fat diet (HFD) induced obesity (DIO) mouse model and administered ZBO by oral gavage from weeks 9 to 12 (Figure 1A). As shown in Figure 1B, there were no significant differences in baseline body weight among the three groups. Compared with the NC group, the DIO group exhibited a sustained and significant increase in body weight throughout the experimental period. Notably, after 4 weeks of ZBO intervention, body weight gain in the DIO+ZBO group was significantly suppressed, and the final body weight was markedly lower than that in the DIO group, consistent with the gross appearance of the mice (Figure 1C). Subsequent analyses of organ and adipose tissue indices showed that, relative to the NC group, DIO mice displayed significant increases in liver, spleen, white adipose tissue (WAT), and brown adipose tissue (BAT) weights, together with a significant shortening of colon length (Figure 1D–F). ZBO intervention was associated with attenuation of these HFD-induced abnormalities. Compared with the DIO group, hepatomegaly and splenomegaly were markedly ameliorated in the DIO+ZBO group, with significant reductions in liver and spleen weights. In addition, the shortened colon observed in DIO mice was partially restored following ZBO treatment. With respect to adipose tissue, both WAT and BAT weights were significantly lower in the DIO+ZBO group than in the DIO group. Collectively, these results are consistent with the idea that ZBO intervention was associated with attenuation of HFD-induced obesity and associated organ abnormalities in mice, suggesting a beneficial effect on established obesity.

### 3.2. ZBO Improved Impaired Glucose Tolerance and Dyslipidemia in DIO Mice

**Figure 2:**
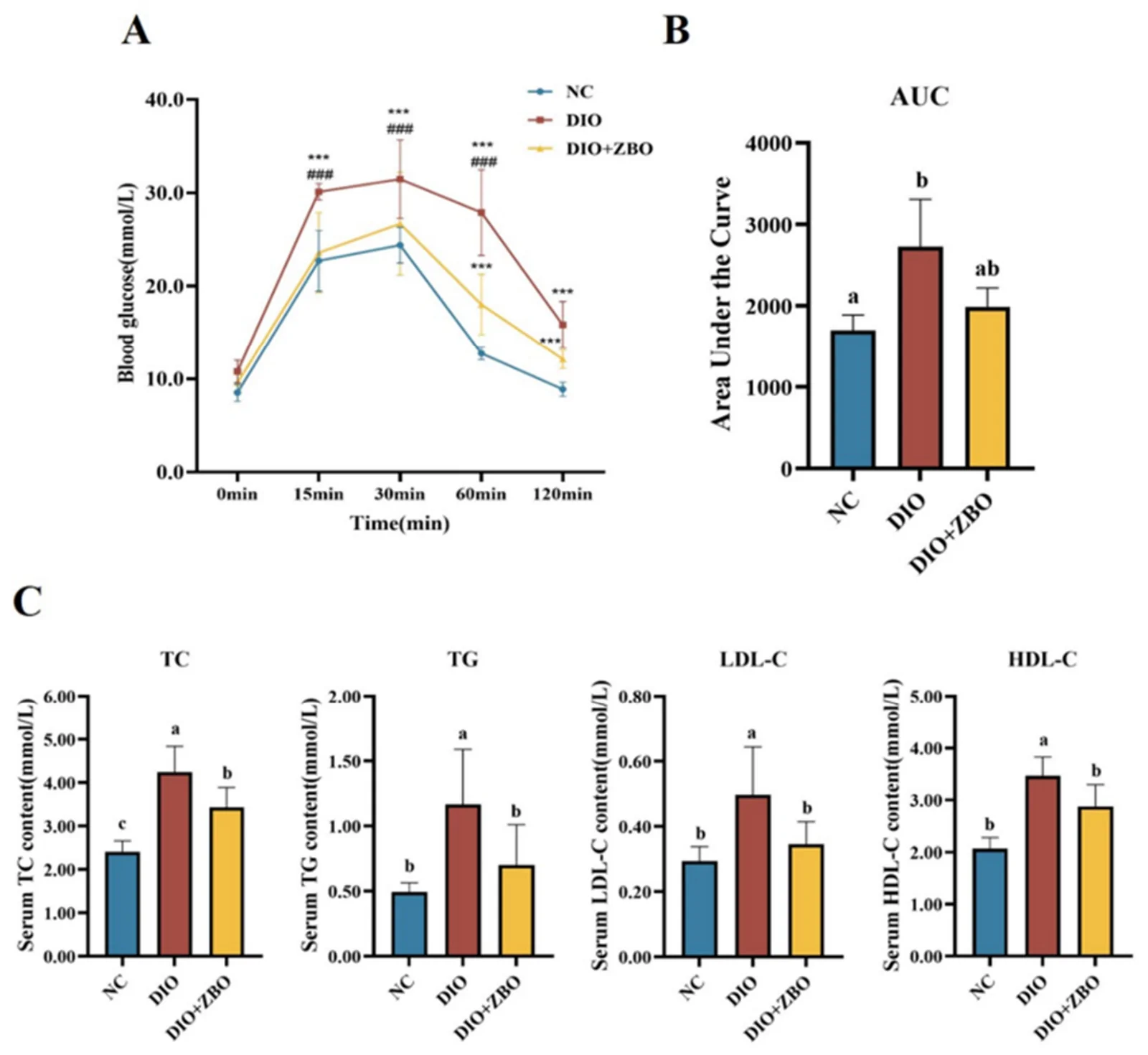
Ameliorative effects of ZBO on glucose tolerance and serum lipid metabolism disorders in DIO mice. (**A**) Glucose tolerance test curves. (**B**) Area under the curve for the glucose tolerance test. (**C**) Serum biochemical parameters of the mice in each group, including total cholesterol, triglycerides, low-density lipoprotein cholesterol, and high-density lipoprotein cholesterol. Data are expressed as the mean ± standard deviation. Different lowercase letters indicate significant differences between groups (*p* < 0.05). Compared with the NC group: ***: *p* < 0.001. Compared with the DIO group: ###: *p* < 0.001.

Assessment of glucose homeostasis was carried out via a glucose tolerance test (GTT) to characterize the metabolic benefits of ZBO. Following an oral glucose challenge, blood glucose levels in the DIO group spiked rapidly, reaching a peak at 30 min that was substantially higher than that of the NC group, and remained elevated throughout the 120 min test period. In contrast, ZBO intervention significantly attenuated this glycemic response; glucose levels in the DIO+ZBO group were consistently lower than those in untreated DIO mice at all time points after 15 min (Figure 2A). This improvement was further confirmed by the area under the curve (AUC) analysis (Figure 2B). The DIO group exhibited a markedly enlarged AUC compared to the NC group, whereas ZBO treatment significantly reduced the AUC, confirming the restoration of glucose tolerance. Furthermore, serum lipid profiles were analyzed to assess lipid metabolism. High-fat diet feeding induced severe dyslipidemia in the DIO group, characterized by significantly elevated serum levels of total cholesterol (TC), triglycerides (TG), low-density lipoprotein cholesterol (LDL-C), and high-density lipoprotein cholesterol (HDL-C) (Figure 2C). Notably, ZBO administration comprehensively reversed these elevations. The levels of all measured lipid parameters in the DIO+ZBO group were significantly lower than those in the DIO group, demonstrating that ZBO effectively ameliorates HFD-induced dyslipidemia.

### 3.3. ZBO Was Associated with Reduced Hepatic Steatosis and Liver Injury in DIO Mice

**Figure 3:**
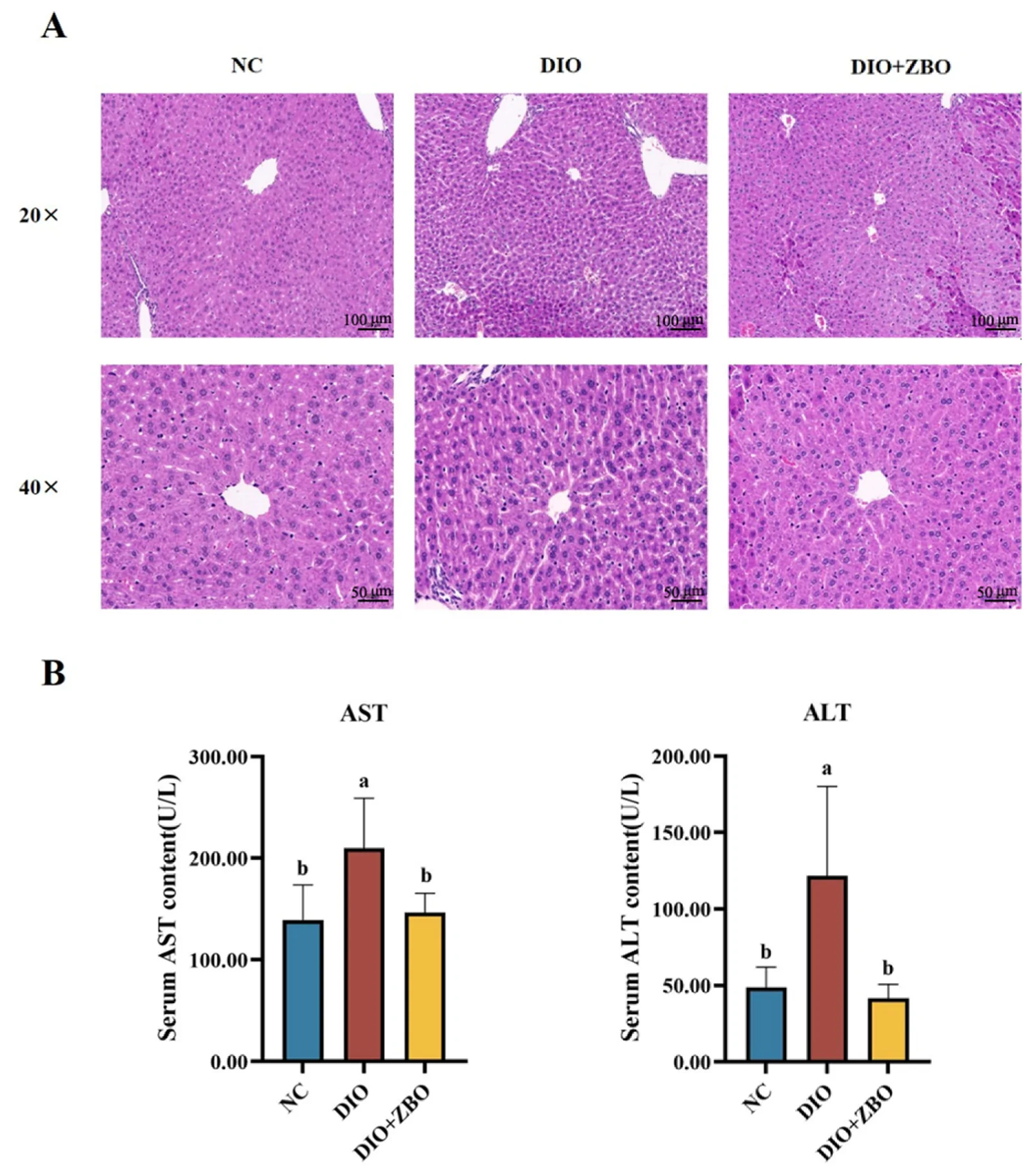
Ameliorative effects of ZBO on hepatic pathological injury and dysfunction in DIO mice. (**A**) Representative H&E staining images of liver tissues from each group. (**B**) Serum hepatic function parameters, including the levels of aspartate aminotransferase and alanine aminotransferase. Data are expressed as the mean ± standard deviation. Different lowercase letters indicate significant differences between groups (*p* < 0.05).

Hematoxylin and eosin (H&E) staining of liver tissue showed that hepatocytes in NC mice were arranged regularly, with clear hepatic lobular architecture and no obvious steatosis, inflammatory infiltration, or hepatocellular necrosis (Figure 3A). In contrast, livers from DIO mice exhibited pronounced vacuolar steatosis, disorganized hepatocyte arrangement, obscured lobular architecture, hepatocellular swelling, and inflammatory cell infiltration. These pathological alterations were markedly ameliorated in the DIO+ZBO group, as evidenced by reduced steatosis, better-preserved lobular architecture, and decreased inflammatory infiltration. Serum biochemical analyses further showed (Figure 3B) that, compared with the NC group, serum aspartate aminotransferase (AST) and alanine aminotransferase (ALT) levels were significantly elevated in DIO mice, indicating substantial HFD-induced hepatocellular injury and liver dysfunction. Compared with the DIO group, serum AST and ALT levels were significantly reduced in the DIO+ZBO group and returned to levels that were not significantly different from those of the NC group. Collectively, these results suggest that ZBO intervention effectively ameliorated HFD-induced hepatic pathological injury and liver dysfunction in obese mice.

### 3.4. ZBO Was Associated with Alterations in the Serum Untargeted Metabolomic Profile and Key Metabolic Pathways in DIO Mice

**Figure 4:**
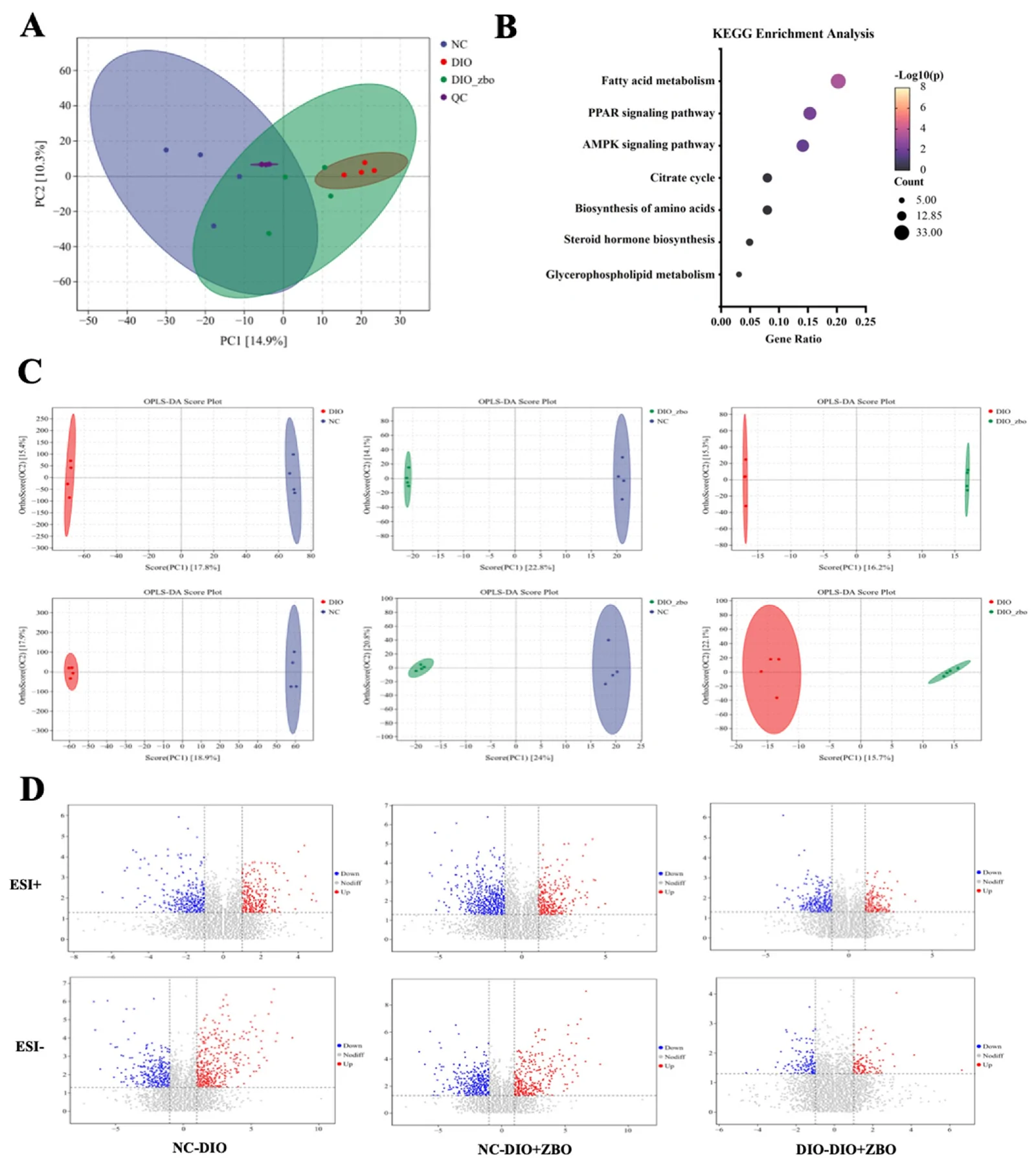
Effects of ZBO on the serum metabolome of DIO mice. (**A**) Principal component analysis score plot of the serum metabolome. (**B**) KEGG pathway enrichment bubble plot of the differential metabolites. (**C**) Orthogonal partial least squares-discriminant analysis score plots for different comparison groups. (**D**) Volcano plots of the differential metabolites across different comparison groups. Data are expressed as the mean ± standard deviation.

Untargeted metabolomic analysis was performed on serum samples from the three groups. Principal component analysis (PCA) showed a clear trend toward separation among the NC, DIO, and DIO+ZBO groups in multivariate space (Figure 4A). In particular, the DIO group was clearly separated from the NC group, indicating that the HFD markedly altered the serum metabolic composition of mice. Relative to the DIO group, samples from the DIO+ZBO group shifted markedly toward the NC group, suggesting that ZBO partially reversed obesity-associated metabolomic alterations. Moreover, the tight clustering of quality control samples indicated good reproducibility of the analytical data. Orthogonal partial least squares discriminant analysis (OPLS-DA) further demonstrated clear intergroup separation across all comparisons (Figure 4C), with good model predictability, indicating the presence of distinct discriminatory metabolites among the three groups. Volcano plot analysis based on OPLS-DA-screened metabolites showed (Figure 4D) that, under combined positive-ion (ESI+) and negative-ion (ESI−) modes, 119 metabolites were upregulated and 65 were downregulated in the DIO group relative to the NC group, whereas 114 metabolites were upregulated and 99 were downregulated in the DIO+ZBO group relative to the NC group. Compared with the DIO group, the DIO+ZBO group exhibited 39 upregulated and 70 downregulated metabolites. To further identify differentially accumulated metabolites, 154 metabolites were screened using Variable Importance in Projection (VIP) ≥ 1.5 and *p* < 0.05 as the selection criteria (Table S3). KEGG pathway enrichment analysis showed that these differential metabolites were mainly enriched in key metabolic pathways, including fatty acid metabolism, the PPAR-α signaling pathway, the AMPK signaling pathway, the tricarboxylic acid cycle, amino acid biosynthesis, and glycerophospholipid metabolism, with fatty acid metabolism showing the highest degree of enrichment (Figure 4B). Overall, ZBO intervention was associated with marked changes in the serum metabolomic profile of HFD-induced obese mice, with the most prominent alterations observed in pathways related to fatty acid metabolism and energy metabolism.

### 3.5. ZBO Was Associated with Altered mRNA Expression of Lipid Metabolism-Related Genes

**Figure 5:**
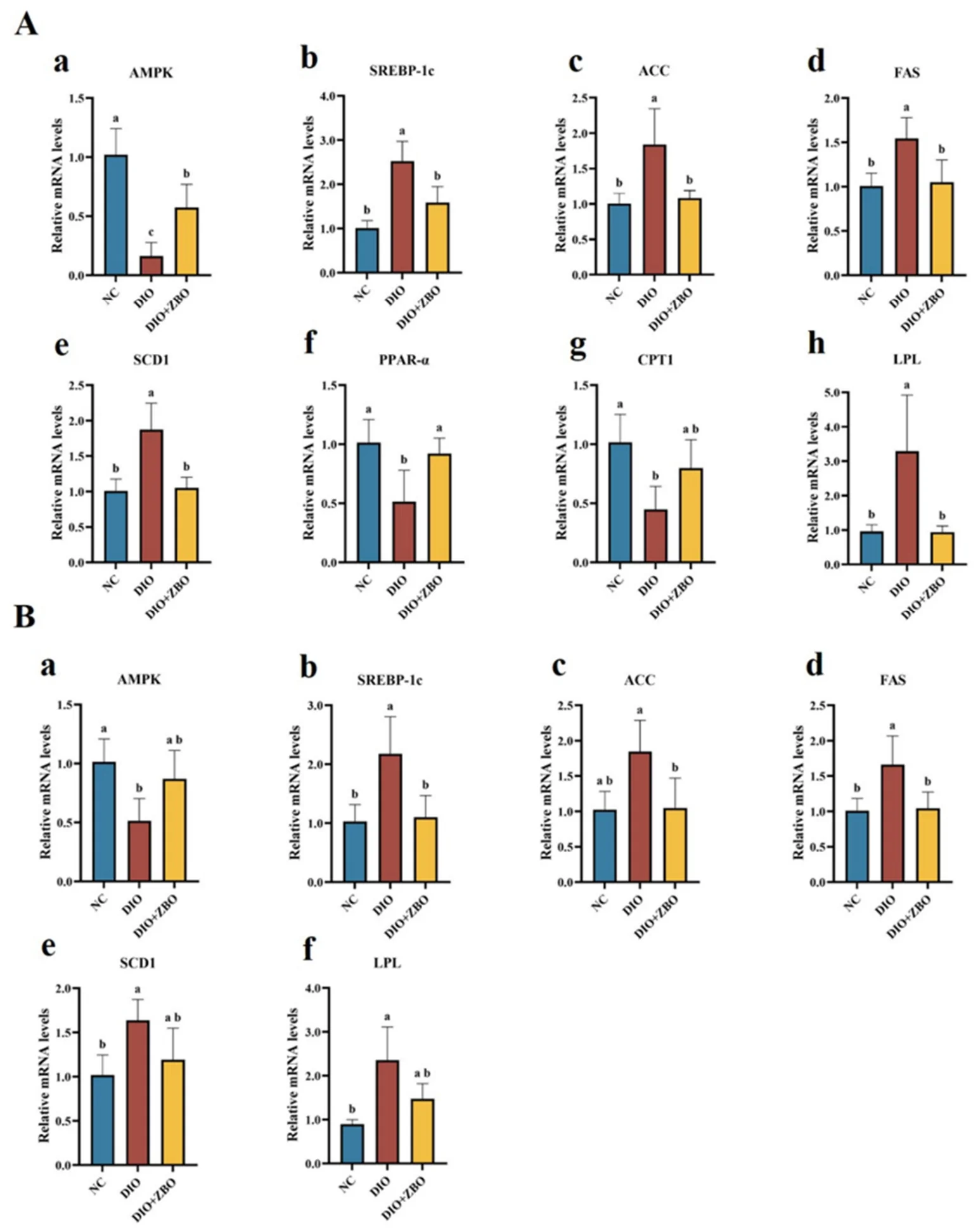
Effects of ZBO on the expression of genes associated with fatty acid metabolism in the liver and adipose tissues of DIO mice. (**A**) Relative mRNA expression levels of key genes involved in fatty acid metabolism in the livers across groups: (**a**) AMPK, (**b**) PPAR‑α, (**c**) CPT1, (**d**) SREBP‑1c, (**e**) ACC, (**f**) FAS, (**g**) SCD1, and (**h**) LPL. (**B**) Relative mRNA expression levels of key genes involved in lipid metabolism in the adipose tissues across groups: (**a**) AMPK, (**b**) SREBP‑1c, (**c**) ACC, (**d**) FAS, (**e**) SCD1, and (**f**) LPL. Data are expressed as the mean ± standard deviation. Different lowercase letters above the bars indicate significant differences between groups (*p* < 0.05)

The mRNA expression levels of key genes involved in fatty acid metabolism within liver and adipose tissues were quantified, providing robust molecular validation for how ZBO ameliorates lipid disorders. In liver tissue (Figure 5A), compared with the NC group, DIO mice showed significant downregulation of AMP-activated protein kinase (AMPK), peroxisome proliferator-activated receptor-α (PPAR-α), and carnitine palmitoyl transferase 1 (CPT1), together with significant upregulation of lipogenic genes, including sterol regulatory element-binding protein 1c (SREBP-1c), acetyl-CoA carboxylase (ACC), fatty acid synthase (FAS), stearoyl-CoA desaturase 1 (SCD1), and lipoprotein lipase (LPL). Similarly, in adipose tissue, AMPK mRNA expression was significantly reduced, whereas SREBP-1c, ACC, FAS, SCD1, and LPL were significantly increased in DIO mice (Figure 5B), indicating systemic dysregulation of lipid metabolism characterized by excessive activation of fatty acid synthesis and impaired oxidative metabolism in both the liver and adipose tissue. Following ZBO intervention, hepatic expression of AMPK, PPAR-α, and CPT1 was significantly increased in the DIO+ZBO group relative to the DIO group, whereas the expression of SREBP-1c, ACC, FAS, SCD1, and LPL was significantly decreased. In adipose tissue, AMPK mRNA expression was also significantly increased, while SREBP-1c, ACC, FAS, SCD1, and LPL were significantly downregulated. These results indicate that ZBO administration was associated with altered expression of key genes involved in fatty acid synthesis and oxidation in both liver and adipose tissue, consistent with the metabolomic findings.

### 3.6. ZBO Was Associated with Changes in Gut Microbial Diversity and Community Structure in DIO Mice

**Figure 6:**
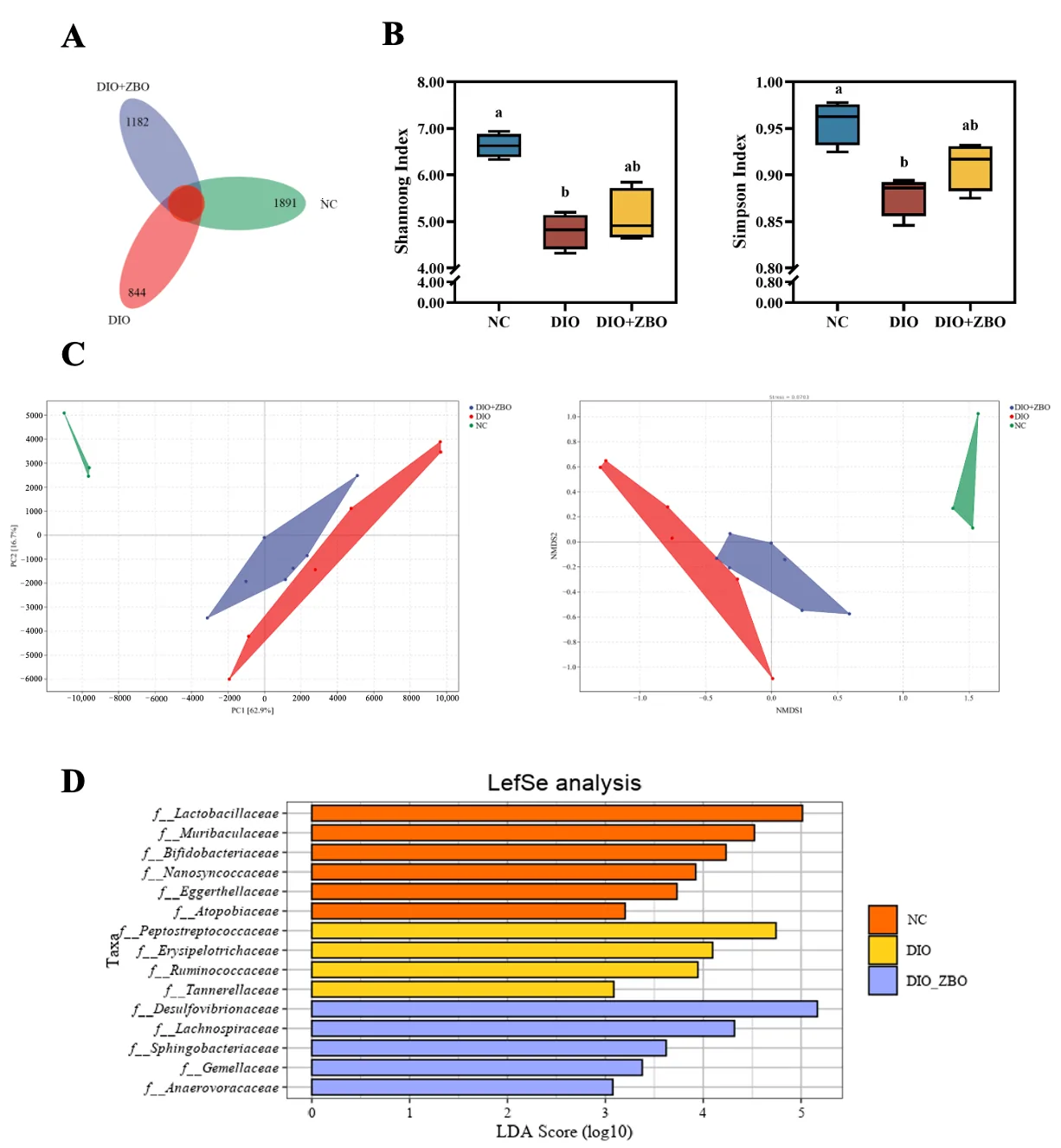
Regulatory effects of ZBO on the diversity and structural composition of the gut microbiota in DIO mice. (**A**) Venn diagram of the operational taxonomic units of the gut microbiota across groups. (**B**) Alpha diversity of the gut microbiota, including the Shannon and Simpson indices. (**C**) Beta diversity of the gut microbiota, including principal coordinate analysis and non-metric multidimensional scaling scatter plots. (**D**) Linear discriminant analysis (LDA) effect size score plot of the differential microbiota (LDA score > 3). Data are expressed as the mean ± standard deviation. Different lowercase letters indicate significant differences between groups (*p* < 0.05).

16S rRNA gene sequencing was employed to elucidate how ZBO alters the gut microbial composition in obese mice. The Venn diagram showed (Figure 6A) that the NC, DIO, and DIO+ZBO groups contained 1891, 844, and 1182 unique ASVs, respectively, with 289 ASVs shared among all three groups. These findings indicate that the HFD markedly reduced gut microbial richness, whereas ZBO intervention partially restored ASV richness. Alpha-diversity analysis showed (Figure 6B) that the Shannon and Simpson indices were both significantly lower in the DIO group than in the NC group, indicating reduced gut microbial diversity following HFD feeding. In contrast, both indices were significantly increased in the DIO+ZBO group relative to the DIO group, suggesting that ZBO was associated with increased gut microbial diversity. Principal coordinates analysis (PCoA) and non-metric multidimensional scaling (NMDS) further revealed clear separation among the NC, DIO, and DIO+ZBO groups (Figure 6C). Samples from the DIO group were located far from those of the NC group, whereas samples from the DIO+ZBO group shifted markedly toward the NC group relative to the DIO group, indicating that the HFD substantially altered the overall structure of the gut microbiota and that ZBO intervention effectively remodeled microbial community structure in obese mice. Linear discriminant analysis effect size (LEfSe) analysis (LDA score > 3; Figure 6D) identified distinct discriminatory taxa among the three groups. The NC group was mainly enriched in recognized beneficial bacterial families such as Lactobacillaceae and Bifidobacteriaceae, which play important roles in maintaining intestinal microbial homeostasis. In contrast, the DIO group was significantly enriched in potentially pro-inflammatory taxa or taxa associated with metabolic disorders, including Peptostreptococcaceae and Erysipelotrichaceae. The DIO+ZBO group showed significant enrichment of taxa such as Lachnospiraceae. Taken together, these results indicate that ZBO intervention significantly ameliorated the HFD-induced reduction in gut microbial diversity and the associated disruption of community structure.

### 3.7. ZBO Was Associated with Changes in Gut Microbiota Composition in DIO Mice

**Figure 7:**
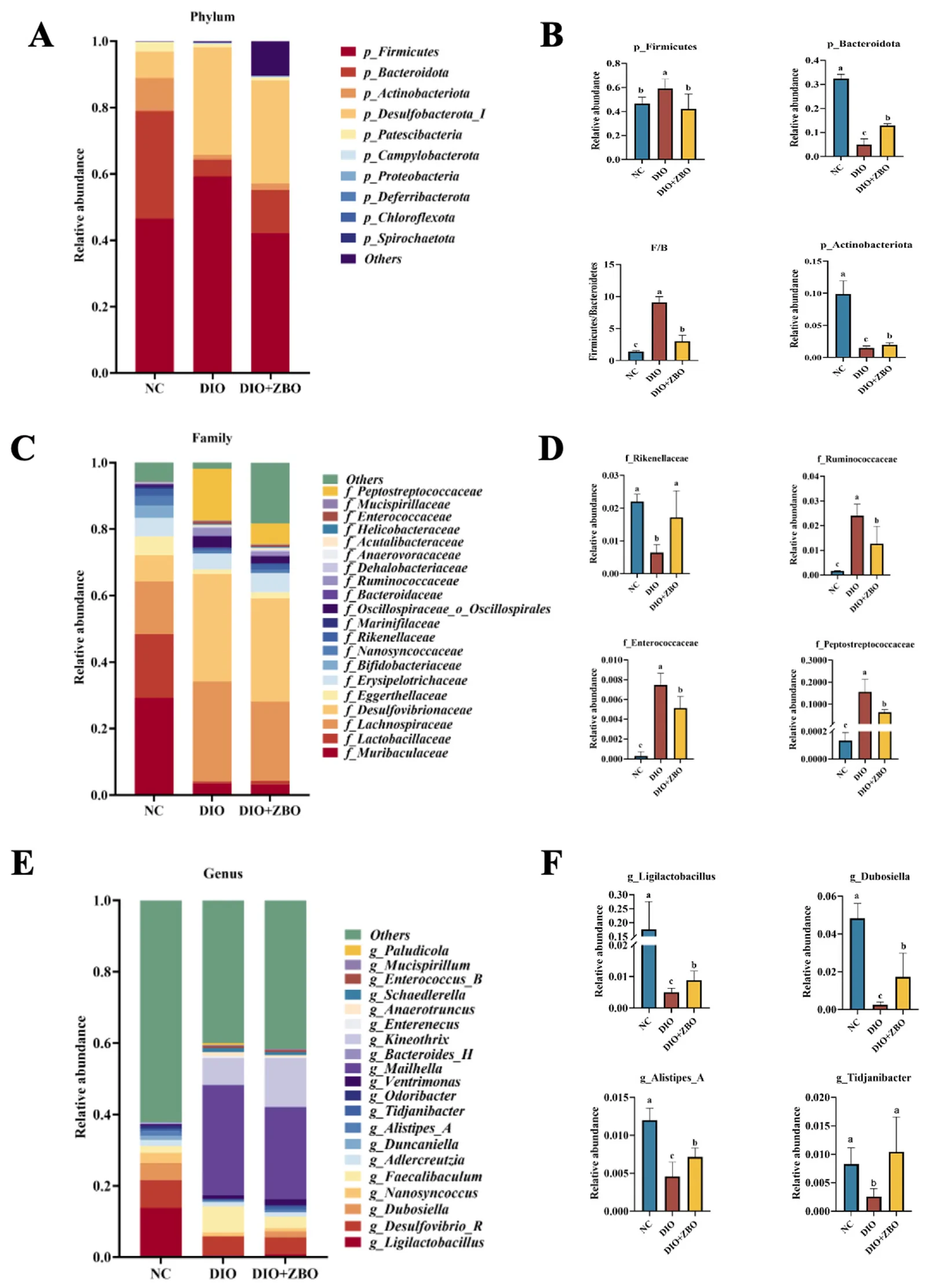
Effects of ZBO on the relative abundances of the gut microbiota at different taxonomic levels in DIO mice. (**A**,**C**,**E**) Stacked bar charts showing the relative abundances of the gut microbiota at the phylum, family, and genus levels across groups. (**B**,**D**,**F**) Bar charts displaying the relative abundances of representative microbiota at the phylum, family, and genus levels. Data are expressed as the mean ± standard deviation. Different lowercase letters indicate significant differences between groups (*p* < 0.05).

The taxonomic composition of the gut microbiota was further profiled at the phylum, family, and genus levels to elucidate the regulatory effects of ZBO. Firmicutes, Bacteroidota, and Actinobacteriota constituted the dominant phyla across all groups (Figure 7A,B). High-fat diet feeding induced a typical obesity-associated microbial signature in the DIO group, characterized by a marked depletion of Bacteroidota and Actinobacteriota, alongside an enrichment of Firmicutes and a consequently elevated Firmicutes-to-Bacteroidota (F/B) ratio. Notably, ZBO treatment effectively counteracted these shifts, restoring the abundance of these major phyla and normalizing the F/B ratio to levels comparable to the NC group. Similar restorative effects were observed at the family level (Figure 7C,D). DIO mice exhibited profound expansions of Ruminococcaceae, Enterococcaceae, and Peptostreptococcaceae, accompanied by a concurrent reduction in Rikenellaceae. Administration of ZBO successfully blunted these pathological alterations. Further classification at the genus level revealed that beneficial taxa, including Ligilactobacillus, Dubosiella, and Alistipes_A, were significantly suppressed in DIO mice (Figure 7E,F). ZBO supplementation not only robustly replenished these beneficial genera but also fine-tuned the abundance of other specific taxa, such as Tidjanibacter. Collectively, these data demonstrate that ZBO systematically remodels the gut microbiota hierarchy, comprehensively mitigating HFD-induced microbial dysbiosis.

### 3.8. Association Network Analysis Revealed Links Between Gut Microbiota and Key Metabolic Signaling Pathways

**Figure 8:**
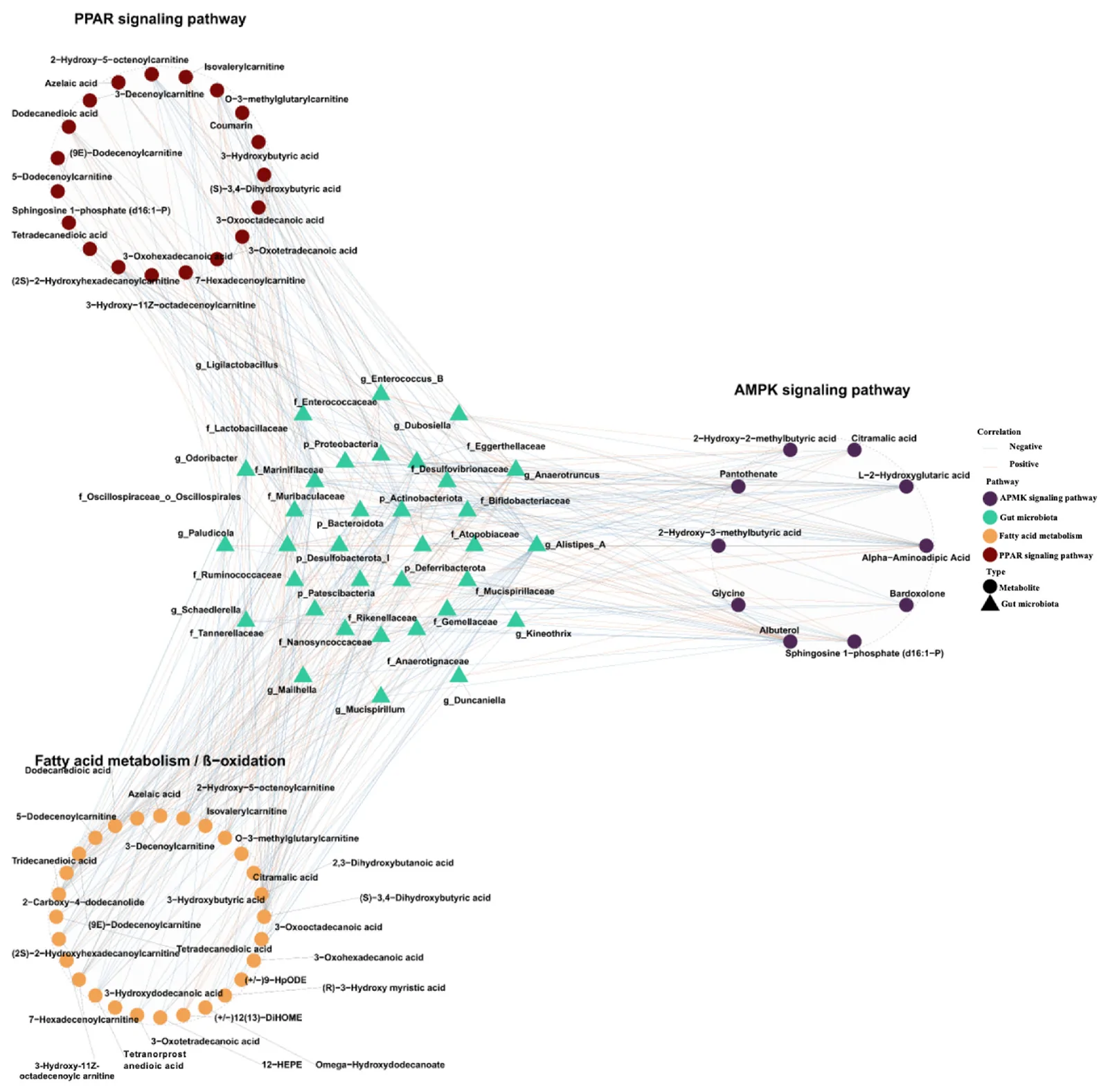
Correlation network between the gut microbiota and key metabolic pathways. The Figure displays the interactions between the gut microbiota and metabolites associated with three key metabolic pathways. Data are expressed as the mean ± standard deviation.

A correlation network linking differential gut microbial taxa to characteristic metabolites in key pathways was constructed to explore correlations between gut microbial taxa and host metabolites (Figure 8). The results revealed extensive and significant correlations between the gut microbiota and multiple key metabolites within these pathways. The metabolites represented in the network were mainly organic acids, acylcarnitines, and fatty acid derivatives, including alpha-aminoadipic acid, 3-hydroxybutyric acid, isovalerylcarnitine, azelaic acid, dodecanedioic acid, tetradecanedioic acid, and 3-oxohexadecanoic acid. Many of the detected acylcarnitines and hydroxy fatty acids represent intermediates in fatty acid β-oxidation. Positive correlations suggested that certain taxa, such as Lactobacillaceae, Bifidobacteriaceae, and Muribaculaceae, may be associated with activation of the AMPK and PPAR signaling pathways by promoting fatty acid catabolism or generating metabolites such as 3-hydroxybutyric acid. In contrast, negative correlations suggested that taxa such as Desulfovibrionaceae and Mucispirillum may be associated with suppression of these pathways by inhibiting fatty acid oxidation or producing pro-inflammatory metabolites. In addition, dicarboxylic acids detected in the network, such as azelaic acid and dodecanedioic acid, as well as molecules such as coumarin and bardoxolone, have been reported to act as PPAR-α ligands or to participate in antioxidant and anti-inflammatory responses. Collectively, these correlational findings suggest associations between ZBO-associated changes in the gut microbiota and alterations in fatty acid metabolism-related metabolites, which in turn were correlated with metabolites linked to AMPK and PPAR-α signaling pathways. These observations raise the hypothesis that the gut microbiota–metabolite axis may be involved in the effects associated with ZBO administration, but this hypothesis requires direct functional testing.

### 3.9. Correlation Analysis of Gut Bacterial Genera and Fatty Acid Metabolites Identified Associations with Key Metabolites

**Figure 9:**
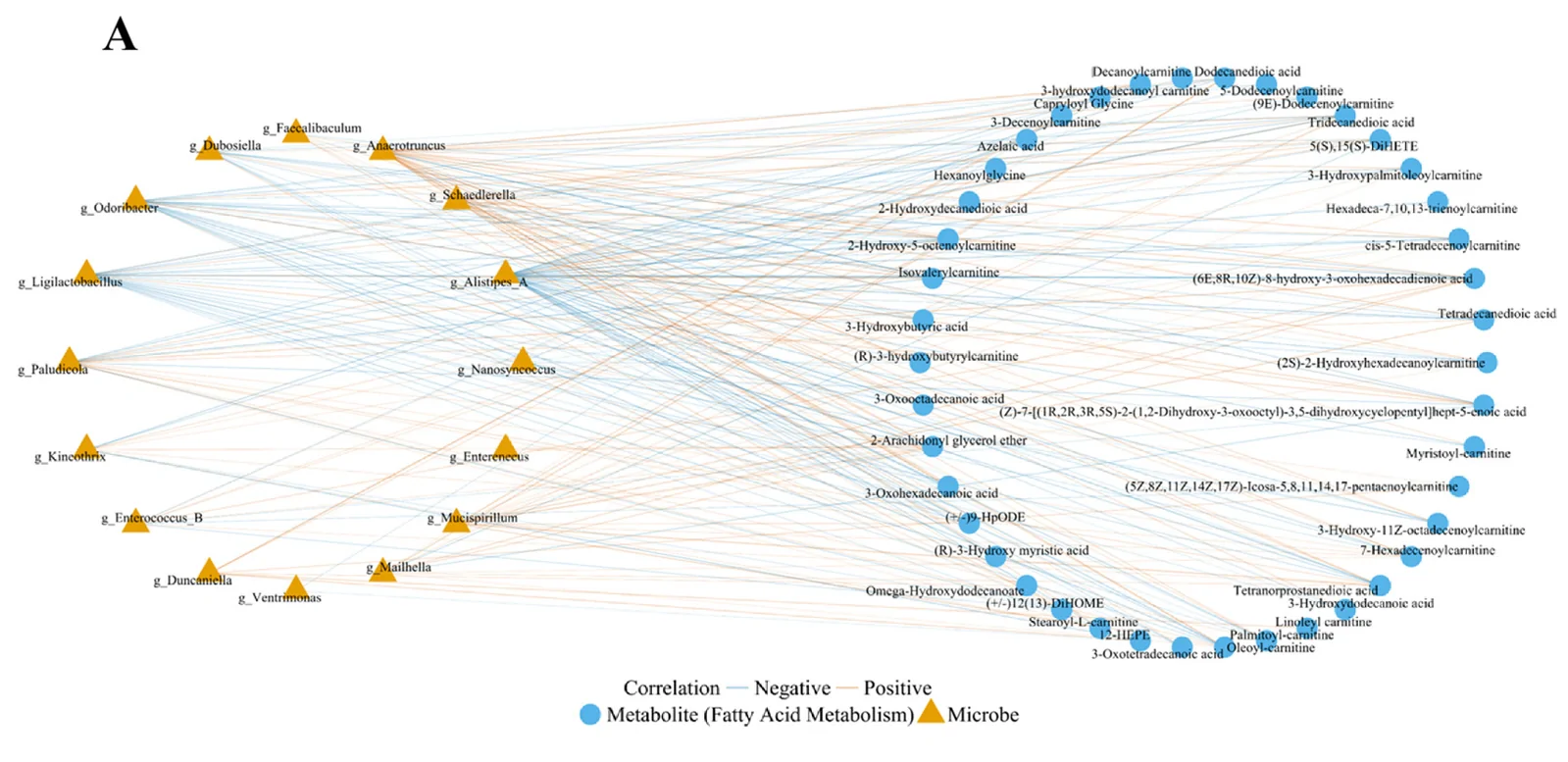

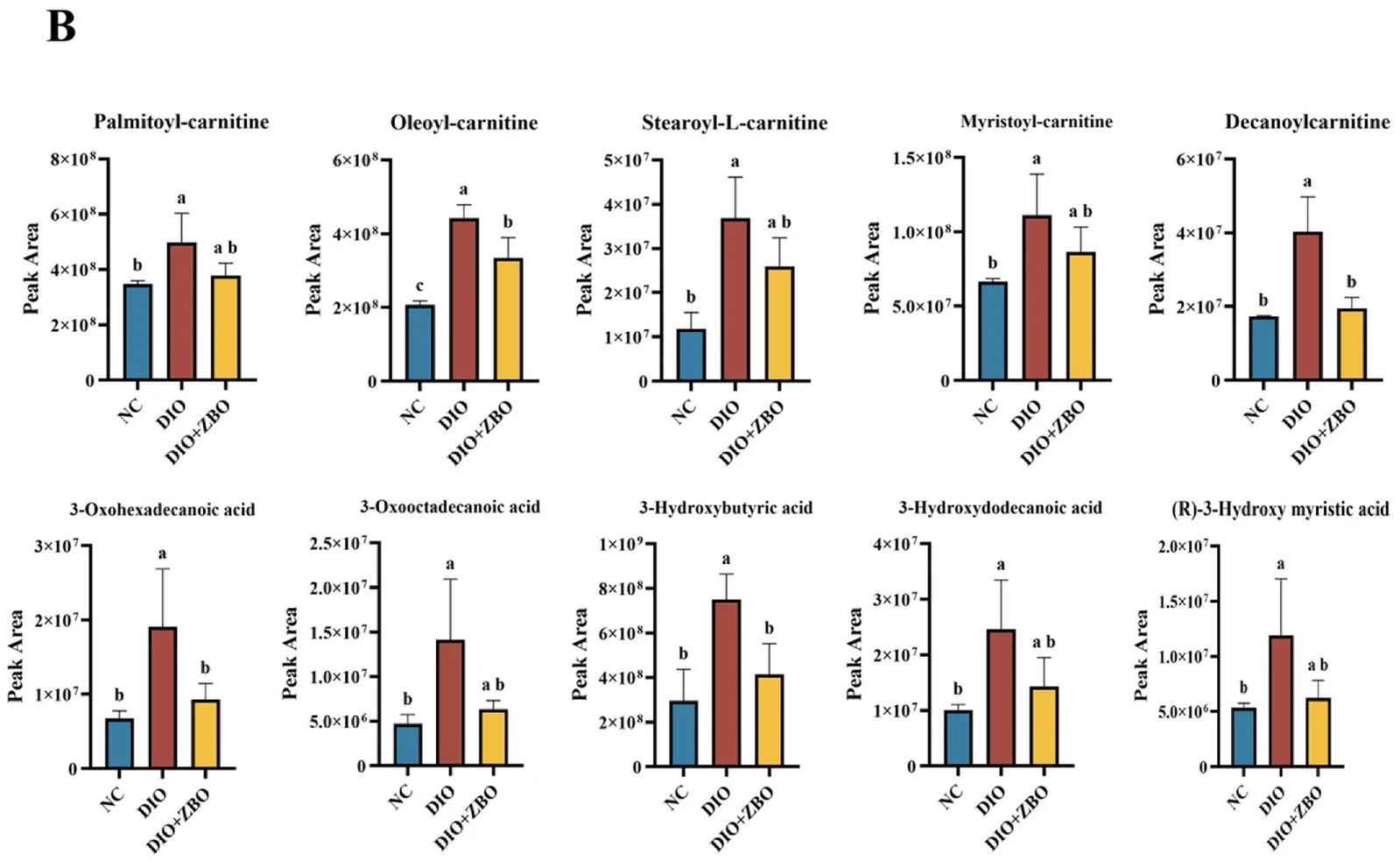
Correlation network between the gut microbiota and fatty acid metabolites, and alterations in the levels of key metabolites. (**A**) Correlation network between the gut microbiota at the genus level and differential metabolites enriched in the fatty acid metabolism pathway (Spearman’s correlation coefficient |r| > 0.6 and *p* < 0.05). (**B**) Relative abundances of the 10 key fatty acid metabolites selected from the network across the NC, DIO, and DIO+ZBO groups (expressed as mass spectrometric peak areas). Data are expressed as the mean ± standard deviation. Different lowercase letters indicate significant differences between groups (*p* < 0.05).

Focusing specifically on the fatty acid metabolic pathway, we constructed a correlation network between genus-level microbiota and differential metabolites to delineate the mechanistic link underlying ZBO’s modulation of dysregulated lipid metabolism (Figure 9A). The results showed that multiple bacterial genera were significantly correlated with key metabolites involved in fatty acid metabolism (Spearman’s |r| > 0.6, *p* < 0.05). Among them, beneficial genera whose abundances were significantly reduced relative to the NC group, such as Ligilactobacillus, Dubosiella, and Alistipes_A, were significantly negatively correlated with fatty acid derivatives including 3-hydroxydodecanoic acid, palmitoyl-carnitine, 3-oxohexadecanoic acid, and 3-oxooctadecanoic acid, whereas several genera enriched in the DIO group were significantly positively correlated with these metabolites. Quantitative analysis of the 10 key fatty acid metabolites identified from the network showed (Figure 9B) that, compared with the NC group, serum levels of acylcarnitine and putative incomplete β-oxidation intermediates represented by 3-hydroxydodecanoic acid were significantly elevated in DIO mice. ZBO intervention markedly reduced the levels of these metabolites, restoring them toward those of the NC group. Overall, these findings reveal associations among ZBO administration, alterations in specific gut bacterial genera, and changes in key fatty acid metabolites, providing a basis for future studies to determine whether these microbial changes contribute functionally to the metabolic effects of ZBO.

## 4. Discussion

In this study, we systematically evaluated the anti-obesity effects of *Zanthoxylum bungeanu m* essential oil (ZBO) in a diet-induced obese mouse model and explored potential correlations through an integrative multi-omics approach encompassing serum metabolomics, gut microbiome profiling, and targeted transcriptomics. Our findings demonstrated that ZBO significantly attenuated HFD-induced body weight gain, visceral adiposity, glucose intolerance, dyslipidemia, and hepatic steatosis. Integrated analyses further revealed that these phenotypic improvements were associated with coordinated changes in serum fatty acid metabolites, remodeling of gut microbial community structure, and altered expression of genes governing fatty acid synthesis and oxidation, particularly those involving AMPK/PPAR-α signaling. Collectively, these data suggest that ZBO administration was associated with systemic changes in lipid metabolism, and that the gut microbiota–fatty acid metabolic axis may represent a relevant nexus for future mechanistic investigation.

Obesity is fundamentally characterized by a chronic mismatch between fatty acid influx, oxidation, and storage. Under HFD conditions, excessive fatty acids enter the liver and adipose tissue; when this influx overwhelms mitochondrial β-oxidation capacity, incomplete oxidation ensues, leading to the accumulation of acylcarnitines and other lipid intermediates [26,27,28]. Our metabolomic data strongly reflected this pathological state. Serum levels of multiple acylcarnitines—including palmitoyl-carnitine, myristoyl-carnitine, oleoyl-carnitine, and decanoylcarnitine—were markedly elevated in DIO mice, suggesting impaired mitochondrial fatty acid transport and inefficient processing of long-chain fatty acyl groups [29,30]. Concurrently, increased levels of β-oxidation intermediates such as 3-hydroxydodecanoic acid, 3-oxohexadecanoic acid, and 3-oxooctadecanoic acid pointed to a metabolic bottleneck wherein fatty acids enter the oxidative pathway but cannot be completely degraded [31,32]. These metabolomic alterations are consistent with the concept of “mitochondrial overload” [26], wherein excessive lipid supply exceeds the oxidative machinery, resulting in the spillover of incompletely oxidized species into the circulation. Notably, ZBO intervention significantly reduced the levels of these acylcarnitines and hydroxy fatty acid intermediates, restoring them toward those observed in NC mice. This normalization suggests that ZBO improves fatty acid utilization efficiency rather than merely reducing lipid supply—a distinction that has important implications for understanding its mechanism of action.

The gene expression data provided complementary molecular evidence for the metabolomic findings. In DIO mice, the hepatic expression of lipogenic genes—*SREBP-1c*, *ACC*, *FAS*, *SCD1*, and *LPL*—was significantly upregulated, while oxidative genes (*AMPK*, *PPAR-α*, and *CPT1*) were suppressed. This pattern indicates a shift toward de novo lipogenesis and away from fatty acid oxidation, a hallmark of obesity-related metabolic dysfunction [8,9]. In adipose tissue, a similar transcriptional profile was observed, suggesting systemic metabolic reprogramming. ZBO treatment was associated with attenuation of these changes in both tissues: oxidative genes were upregulated, and lipogenic genes were downregulated. These coordinated transcriptional responses are noteworthy because they suggest that ZBO acts simultaneously on both fatty acid synthesis and oxidation pathways. The reciprocal regulation of *ACC* (which produces malonyl-CoA, a potent CPT1 inhibitor) and *CPT* (the rate-limiting enzyme for mitochondrial fatty acid uptake) is particularly interesting, as they raise the possibility that ZBO administration might be associated with reduced malonyl-CoA-mediated inhibition of mitochondrial fatty acid oxidation, but this hypothesis requires direct biochemical confirmation [33]. However, it must be emphasized that these conclusions are based on mRNA expression data only. AMPK activity is primarily regulated by phosphorylation at Thr172, and mRNA levels do not necessarily reflect protein activity. Thus, the observed changes in *AMPK* mRNA should be interpreted as transcriptional responses consistent with, but not evidence of, pathway activation. Western blot analysis of p-AMPK/AMPK and p-ACC/ACC ratios is urgently needed to validate whether ZBO indeed activates AMPK signaling at the functional level.

The 16S rRNA sequencing results revealed that HFD feeding profoundly reduced gut microbial diversity, altered community structure, and increased the Firmicutes/Bacteroidota (F/B) ratio—a microbial signature frequently associated with obesity and enhanced energy harvest [34]. Specifically, the DIO group showed significant enrichment of potentially pro-inflammatory taxa such as Peptostreptococcaceae and Erysipelotrichaceae, which have been positively correlated with abdominal adiposity and chronic inflammation in population-level studies [35,36]. Conversely, beneficial genera including *Ligilactobacillus*, *Dubosiella*, and *Alistipes* were markedly depleted in obese mice. ZBO intervention effectively counteracted these changes: it restored overall microbial diversity, normalized the F/B ratio, and increased the abundance of *Alistipes*—a genus previously associated with reduced hepatic triglyceride deposition and improved lipid metabolism [37]. These compositional changes raise the hypothesis that ZBO may improve host metabolism by fostering a gut environment that favors oxidative over lipogenic programming. However, the functional consequences of these compositional shifts remain largely speculative. Correlation analyses between specific bacterial taxa and metabolites revealed numerous significant associations (Spearman’s |r| > 0.6, *p* < 0.05), particularly between *Alistipes*, *Odoribacter*, and acylcarnitine/hydroxy fatty acid metabolites. For instance, the negative correlations of *Alistipes* with 3-hydroxydodecanoic acid and palmitoyl-carnitine suggest a potential link between this genus and improved fatty acid oxidation efficiency. Nevertheless, these associations are purely correlational and do not establish causality. It remains entirely possible that the microbial changes observed are secondary to improvements in host metabolism rather than drivers of those improvements.

The effects of ZBO observed here are comparable to those reported for several well-characterized natural products with anti-obesity properties. Berberine, an isoquinoline alkaloid, has been shown to activate AMPK and modulate the gut microbiota in DIO mice, resulting in reduced body weight and improved glucose tolerance [38,39]. However, berberine’s poor oral bioavailability limits its translational potential, whereas ZBO’s volatile nature may offer distinct pharmacokinetic properties. Resveratrol, a polyphenolic compound, improves lipid metabolism via SIRT1/AMPK pathways but has relatively modest effects on gut microbial diversity compared with the broad compositional remodeling observed with ZBO [40,41]. Capsaicin and green tea catechins exert their anti-obesity effects primarily through thermogenesis and fat oxidation, with less pronounced impacts on fatty acid oxidation intermediates [42,43,44,45]. The unique feature of ZBO appears to be its simultaneous influence on multiple layers—microbial ecology, circulating lipid metabolites, and tissue-level gene expression—suggesting a multi-targeted mode of action. However, direct head-to-head comparisons under identical experimental conditions are lacking, and such comparisons should be interpreted with caution given differences in study design, dosage, and animal models across laboratories.

*Zanthoxylum bungeanum* Maxim. (Huajiao) is a traditional Chinese medicinal and culinary herb listed in the Chinese Pharmacopeia, with a long history of oral consumption. Toxicological studies have reported an oral LD_50_ of 2.27 g/kg in mice, providing a safety margin exceeding 56-fold for the 40 mg/kg dose used in this study [46]. In vitro studies have also shown low cytotoxicity in human cell lines, and trace element analysis has revealed negligible levels of toxic heavy metals [46]. However, several safety gaps must be acknowledged. Only acute toxicity data are currently available; subchronic and chronic toxicity studies have not been performed. The present study assessed liver function via serum ALT/AST levels, but comprehensive histopathological evaluation of other major organs (kidney, heart, spleen, lung) was not conducted. Regarding translational potential, the theoretical human equivalent dose (HED), calculated based on body surface area conversion (mouse Km = 3, human Km = 37), is approximately 3.24 mg/kg, corresponding to ~194 mg/day for a 60 kg adult. While this falls within the range of daily *Zanthoxylum* consumption as a culinary spice, it must be emphasized that this HED is a purely theoretical estimate based on allometric scaling. It does not account for interspecies differences in pharmacokinetics, bioavailability, metabolism, or tissue distribution. Moreover, the present study used a single fixed dose, and no dose–response relationship was established. Therefore, the HED should not be interpreted as a recommended human dose; rigorous dose-ranging and pharmacokinetic studies in higher animal models, followed by systematic clinical trials, are essential before any translational inference can be made. Consequently, ZBO may have potential as a dietary supplement for obesity management, although further dose-ranging, safety, and clinical studies are needed to establish its translational applicability.

## 5. Study Limitations and Future Directions

Several limitations should be considered when interpreting the present findings. The use of a single fixed dose (40 mg/kg) and a 4-week intervention period, together with a sample size of six per group, means that our findings are representative only of the specific experimental conditions tested. Consequently, no conclusions can be drawn regarding dose-dependent effects, sustained efficacy over extended administration, or the possible development of tolerance. Although our multi-omics data reveal strong and consistent associations among gut microbiota changes, fatty acid metabolite alterations, and lipid metabolism-related gene expression, these findings are inherently correlational rather than causal. It remains unclear whether gut microbiota remodeling plays a mediating role in the anti-obesity effects of ZBO, or whether the observed microbial changes are secondary to improvements in host metabolism. Fecal microbiota transplantation (FMT) or microbiota depletion experiments are required to establish causality. While such experiments are beyond the scope of the current study, they are planned as a top priority in our future research to directly validate the ‘gut microbiota–fatty acid metabolism’ axis. Furthermore, functional metagenomic prediction (e.g., PICRUSt2) was not performed, and key microbial metabolites such as short-chain fatty acids (SCFAs) and secondary bile acids were not quantified in cecal content or feces. These analyses would have strengthened the link between ZBO-induced taxonomic shifts and functional metabolic changes in the gut microbiome. The absence of these data limits our ability to fully interpret the functional relevance of the observed microbial remodeling. Therefore, the mechanistic hypotheses linking gut microbiota to host fatty acid metabolism should be considered as provisional and requiring further validation through functional metagenomic and targeted metabolomic approaches in future studies. Metabolite identifications are based on untargeted LC-MS with putative annotation (MSI Level 2), necessitating targeted validation with authentic standards. Similarly, our conclusions regarding AMPK/PPAR pathway engagement rely solely on mRNA expression, whereas functional activation requires protein-level phosphorylation and activity assays (e.g., Western blot for p-AMPK/AMPK and p-ACC/ACC). Safety data remain incomplete, as only acute oral LD_50_ is available; subchronic toxicity, organ-specific histopathology beyond the liver, and reproductive or genotoxic effects have not been evaluated. The human equivalent dose (HED ≈ 3.24 mg/kg) is a theoretical estimate derived from body surface area conversion, and systematic safety and toxicological evaluations have not been performed. Therefore, the present findings should not be directly extrapolated to human application and require further validation. Finally, the observed correlations between specific bacterial genera and acylcarnitine/hydroxy fatty acid metabolites, while statistically significant, offer no direct mechanistic evidence and may be confounded by host physiology or indirect microbial interactions. Future investigations should prioritize dose-ranging and extended-duration studies, FMT-based causal testing, targeted metabolomics with internal standards, protein-level pathway validation, and comprehensive subchronic toxicological evaluation to determine whether ZBO holds genuine therapeutic potential.

## 6. Conclusions

Collectively, these multi-omics data reveal that ZBO administration is associated with remodeling of the gut microbiota, alterations in serum fatty acid metabolites, and changes in the expression of genes involved in lipid metabolism. These coordinated changes provide correlational evidence supporting the potential of ZBO as a dietary supplement strategy for alleviating obesity-related metabolic disorders. Furthermore, the present findings are derived from a mouse model and do not constitute evidence of efficacy or safety in humans. Therefore, the potential application of ZBO as a dietary supplement warrants further investigation through comprehensive preclinical safety evaluation and well-controlled clinical trials.

## Data Availability

The original contributions presented in this study are included in the article/Supplementary Material. Further inquiries can be directed to the corresponding authors.

## Supplementary Materials

The following supporting information can be downloaded at: https://www.mdpi.com/article/10.3390/nu18182950/s1. Figure S1. GC-MS analysis of ZBO and mass spectrometry analysis of linalool and hexanoic acid. Figure S2. Structural formulae of the top ten target compounds by relative content in the ZBO components. Figure S3. Food intake of DIO and DIO+ZBO mice. Table S1. Chemical composition of ZBO. Table S2. Gene names and primer sequences. Table S3. Differential serum metabolites identified by LC-MS. (NC1~ZBO4 ×10^6^ AU).

## Abbreviations

ACC	Acetyl-CoA carboxylase
ALT	Alanine aminotransferase
AMPK	AMP-activated protein kinase
AST	Aspartate aminotransferase
ASVs	Amplified Sequence Variants
CPT1	Carnitine palmitoyltransferase 1
DIO	Diet-induced obese
FAS	Fatty acid synthase
FXR	Farnesoid X receptor
GTT	Glucose tolerance test
HDL-C	High-density lipoprotein cholesterol
HFD	High-fat diet
LDL-C	Low-density lipoprotein cholesterol
LPL	Lipoprotein lipase
PPAR-α	Peroxisome proliferator-activated receptor α
RT-qPCR	Real-time quantitative polymerase chain reaction
SCD1	Stearoyl-CoA desaturase 1
SREBP-1c	Sterol regulatory element-binding protein 1c
TC	Total cholesterol
TG	Triglycerides
TGR5	Takeda G protein-coupled receptor 5
TLR4/NF-κB	Toll-like receptor 4/nuclear factor kappa-B
ZB	*Zanthoxylum bungeanum*
ZBO	*Zanthoxylum bungeanum* essential oil
