# Supplementary material for: Zanthoxylum bungeanum Essential Oil Alleviates Obesity and Dysregulated Lipid Metabolism in Diet-Induced Obese Mice: A Multi-Omics Study Linking Gut Microbiota to Fatty Acid Metabolism
Source: Nutrients. 2026 Sep 9;18(18):2950. doi: 10.3390/nu18182950 (PMC13610182; doi:10.3390/nu18182950)
Supplement: Supplementary file 1 [file nutrients-18-02950-s001.zip › nutrients-4472534-supplementary.pdf]

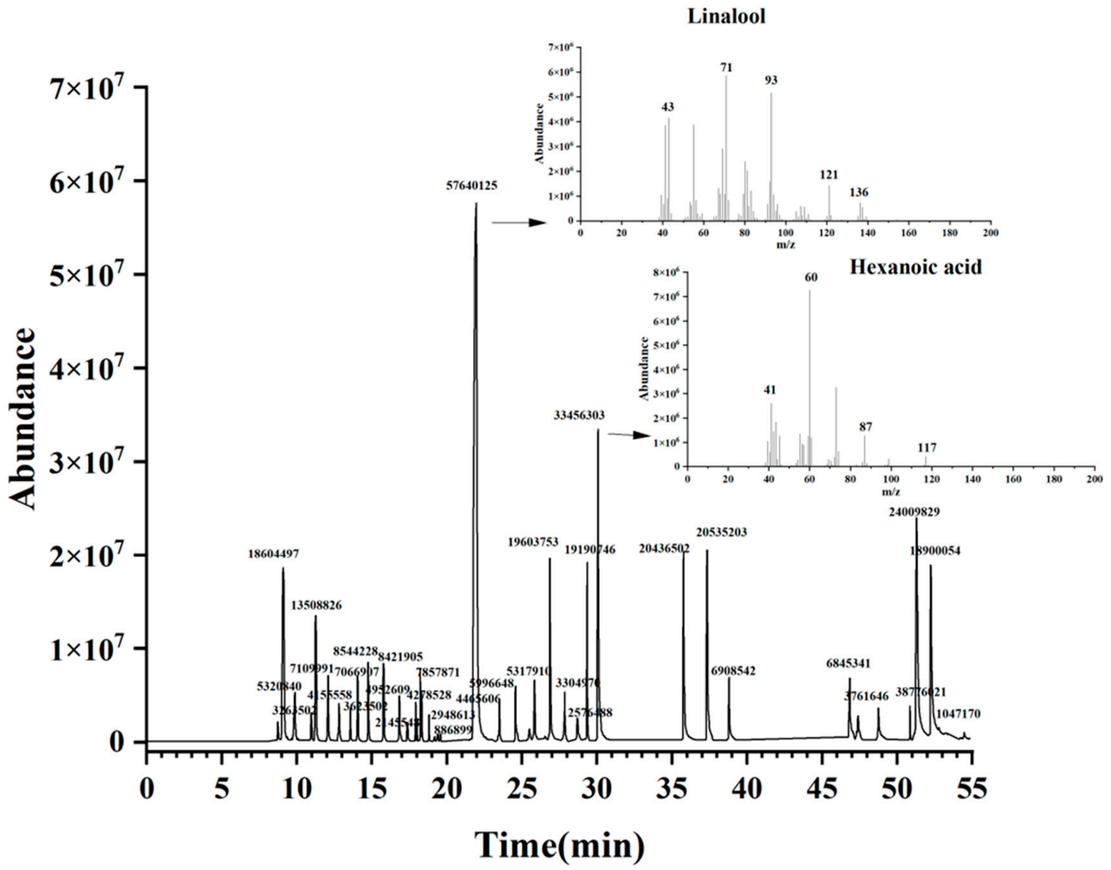

Figure S1. GC-MS analysis of ZBO and mass spectrometry analysis of linalool and hexanoic acid.

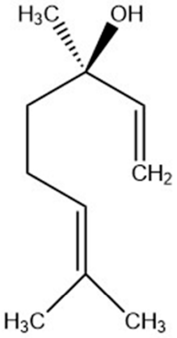

1. Linalool

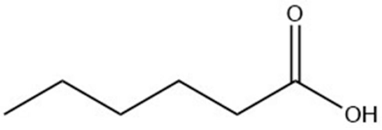

2. Hexanoic acid

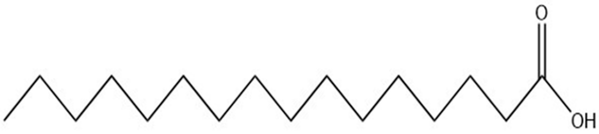

3. Cetylic acid

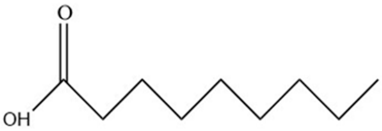

4. Nonanoic acid

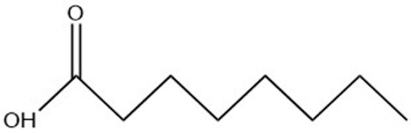

5. Octanoic acid

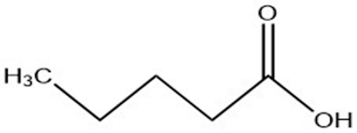

6. Pentanoic acid

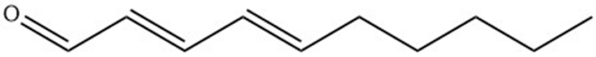

7. trans,trans-2,4-Decadienal

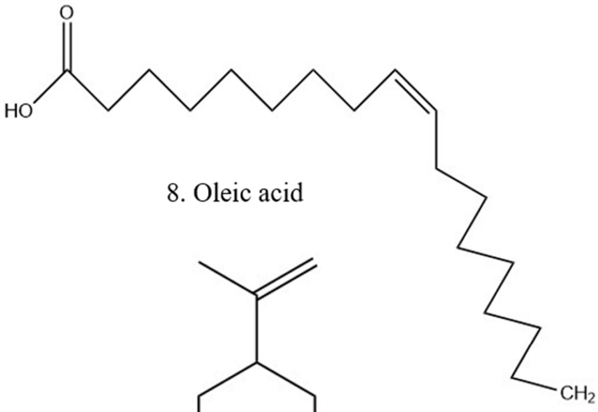

8. Oleic acid

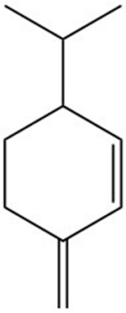

9. β-Phellandrene

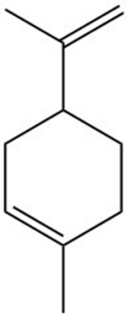

10. D-Limonene

Figure S2. Structural formulae of the top ten target compounds by relative content in the ZBO components.

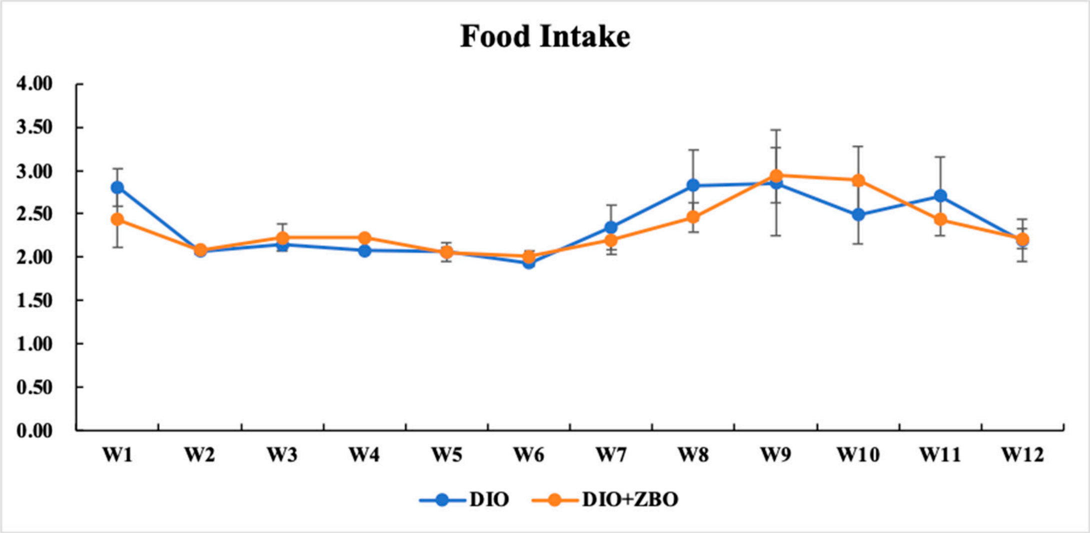

Figure S3. Food Intake of DIO and DIO+ZBO mice.

Table S1. Chemical composition of ZBO.

Number	Compound	Retention time (min)	Relative Content	Molecular Formula	Molecular Weight	CAS Number
**1**	**Linalool**	**21.952**	**16.14%**	$C_{10}H_{18}O$	154	**78-70-6**
**2**	**Hexanoic acid**	**30.081**	**9.37%**	$C_6H_{12}O_2$	**116**	**142-62-1**
**3**	**Cetylic acid**	**51.29**	**6.72%**	$C_{16}H_{32}O_2$	256	**57-10-3**
**4**	**Nonanoic acid**	**37.339**	**5.75%**	$C_9H_{18}O_2$	158	**112-05-0**
**5**	**Octanoic acid**	**35.771**	**5.72%**	$C_8H_{16}O_2$	**144**	**124-07-2**
**6**	**Pentanoic acid**	**26.866**	**5.49%**	$C_5H_{10}O_2$	102	**109-52-4**
**7**	**trans,trans-2,4-Decadienal**	**29.353**	**5.37%**	$C_{10}H_{16}O$	152	**25152-84-5**
**8**	**Oleic acid**	**52.223**	**5.29%**	$C_{18}H_{34}O_2$	282	**112-80-1**
**9**	**β-Phellandrene**	**9.087**	**5.21%**	$C_{10}H_{16}$	136	**555-10-2**
**10**	**D-Limonene**	**11.261**	**3.78%**	$C_{10}H_{16}$	136	**5989-27-5**
**11**	**2,3-Octanedione**	**14.75**	**2.39%**	$C_8H_{14}O_2$	142	**585-25-1**
**12**	**Nonanal**	**15.773**	**2.36%**	$C_9H_{18}O$	142	**124-19-6**
**13**	**Ethanoic acid**	**18.229**	**2.20%**	$C_2H_4O_2$	60	**64-19-7**
**14**	**2-Pentylfuran**	**12.078**	**1.99%**	$C_9H_{14}O$	**138**	**3777-69-3**
**15**	**Octanal**	**14.046**	**1.98%**	$C_8H_{16}O$	**128**	**124-13-0**
**16**	**2-Octenoic acid**	**38.809**	**1.93%**	$C_8H_{14}O_2$	**142**	**1470-50-4**
**17**	**Tetradecanoic acid**	**46.836**	**1.92%**	$C_{14}H_{28}O_2$	228	**544-63-8**
**18**	**(E)-2-Tridecenal**	**24.576**	**1.68%**	$C_{13}H_{24}O$	**196**	**7069-41-2**
**19**	**β-Myrcene**	**9.871**	**1.49%**	$C_{10}H_{16}$	**136**	**123-35-3**
**20**	**α-Terpineol**	**25.856**	**1.49%**	$C_{10}H_{18}O$	**154**	**10482-56-1**
**21**	**(E)-2-Octenal**	**16.834**	**1.39%**	$C_8H_{14}O$	**126**	**2548-87-0**
**22**	**4-Terpineol**	**23.505**	**1.25%**	$C_{10}H_{18}O$	**154**	**562-74-3**
**23**	**Thujone**	**17.927**	**1.20%**	$C_{10}H_{16}O$	**152**	**546-80-5**
**24**	**Ethyl Hexanoate**	**12.8**	**1.16%**	$C_8H_{16}O_2$	144	**123-66-0**
**25**	**4-Methyl-6-Decanol**	**50.856**	**1.09%**	$C_{11}H_{24}O$	172	**213547-15-0**
**26**	**Pentadecanoic acid**	**48.749**	**1.03%**	$C_{15}H_{30}O_2$	242	**1002-84-2**
**27**	**1-Pentanol**	**13.564**	**1.01%**	$C_5H_{12}O$	88	**71-41-0**
**28**	**3-Undecenal**	**27.846**	**0.93%**	$C_{11}H_{20}O$	**168**	**2463-77-6**
**29**	**trans,trans-2,4-Heptandienal**	**18.814**	**0.83%**	$C_7H_{10}O$	110	**4313-03-5**
**30**	**δ-Valerolactone**	**28.692**	**0.72%**	$C_5H_8O_2$	100	**542-28-9**
**31**	**(E)-5-Undecene**	**17.352**	**0.60%**	$C_{11}H_{22}$	**154**	**764-97-6**
**32**	**Eicosanoic acid**	**54.468**	**0.29%**	$C_{20}H_{40}O_2$	312	**506-30-9**
**33**	**Decanal**	**19.555**	**0.23%**	$C_{10}H_{20}O$	156	**112-31-2**

Table S1 presents the chemical composition of ZBO. The 33 most prominent components in ZBO are categorised by retention time and relative abundance, with these parameters revealing the identity and relative abundance of each component.

Table S2. Gene names and primer sequences.

Target gene	Sequence	
**ACC**	**S**	**5'- GACGTTCGCCATAACCAAGT -3'**
	**AS**	**5'- CTGCAGGTTCTCAATGCAAA -3'**
**FAS**	**S**	**5'- CCCTTGATGAAGAGGGATCA-3'**
	**AS**	**5'- ACTCCACAGGTGGGAACAAG -3'**
**LPL**	**S**	**5'-TCCAGAGTTTGACCGCCTTC -3'**
	**AS**	**5'-CCTCAGCTGTGTCTTCAGGG -3'**
**SCD1**	**S**	**5'- AGCTGGTGATGTTCCAGAGG -3'**
	**AS**	**5'- GTGGGCAGGATGAAGCAC-3'**
**CPT1**	**S**	**5'- GAGCCACGAAGCCCTCAAACACAT -3'**
	**AS**	**5'- GCTGTACAACATGGGCTTCCGACCTG -3'**
**AMPK**	**S**	**5'- ACAGAAGCCAAATCAGGGACT-3'**
	**AS**	**5'- CACGGATGAGGTAAGAGAGACT -3'**
**PPAR-α**	**S**	**5'- GGATGTCACACAATGCAATTCGCT -3'**
	**AS**	**5'- TCACAGAACGGCTTCCTCAGGTT -3'**
**SREBP-1c**	**S**	**5'- AAGCAAATCACTGAAGGACCTGG-3'**
	**AS**	**5'- AAAGACAAGGGGCTACTCTGGGAG-3'**
**GAPDH**	**S**	**5'- AGGTCGGTGTGAACGGATTTG -3'**
	**AS**	**5'- TGTAGACCATGTAGTTGAGGTCA -3'**

Table S3. Differential serum metabolites identified by LC-MS. (NC1~ZBO4 $\times 10^6$ AU)

Name	NC1	NC2	NC3	NC4	DIO1	DIO2	DIO3	DIO4	ZBO1	ZBO2	ZBO3	ZBO4	p	vip	RT [min]	m/z	Reference Ion	Formula	HMDB
Alpha-Aminoadipic Acid	9.39	10.15	9.67	8.01	15.19	12.15	16.10	13.64	11.47	12.64	11.93	16.40	0.00	1.61	0.66	160	[M-H]-	C6H11NO4	HMDB0000510
1-Hydroxy-2,2,5,5-tetramethylpyrrolidine-3-carboxylic acid	1.44	1.72	1.09	1.66	4.86	4.51	6.98	7.52	4.48	7.89	8.83	6.68	0.00	1.81	0.67	188	[M+H]+	C9H17NO3	HMDB0243895
N(alpha)-methyl-L-lysine	24.53	27.54	35.77	38.48	9.42	9.26	25.21	8.33	13.46	21.47	11.04	26.44	0.02	1.61	0.67	144	[M+CH3OH+H]+	C7H16N2O2	HMDB0240689
N-Acetyl-1-methylhistidine	5.34	5.13	3.49	4.69	11.65	10.38	9.45	12.04	9.12	10.72	10.18	9.29	0.00	1.89	0.68	212	[M+H]+	C9H13N3O3	HMDB0240340

Continued from the previous Table.

Name	NC1	NC2	NC3	NC4	DIO1	DIO2	DIO3	DIO4	ZBO1	ZBO2	ZBO3	ZBO4	p	vip	RT [min]	m/z	Reference Ion	Formula	HMDB
Levoglucosan	36.88	36.11	35.96	42.45	36.73	33.25	31.06	42.43	24.88	18.22	32.94	27.83	0.02	1.53	0.70	145	[M+CH3OH+H]+	C6H10O5	HMDB0000640
methacholine	243.29	216.26	169.31	166.11	111.14	108.16	100.52	115.09	93.81	139.70	113.75	91.48	0.00	1.73	0.70	160	[M+H]+	C8H18NO2	HMDB0015654
4-(Dimethylamino)butyl carbamimidothioate	28.98	27.25	28.85	24.13	7.20	6.98	7.57	7.64	6.67	6.60	7.16	6.09	0.00	1.90	0.70	176	[M+H]+	C7H17N3S	HMDB0246305
N-gamma-L-Glutamyl-D-alanine	16.81	16.05	13.27	14.92	14.30	6.66	10.39	6.23	11.76	16.26	12.20	10.46	0.04	1.59	0.70	219	[M+H]+	C8H14N2O5	HMDB0036301
(S)-3,4-Dihydroxybutyric acid	48.11	42.08	43.16	40.38	85.48	80.53	82.74	110.94	68.77	73.12	75.24	75.58	0.00	1.84	0.70	119	[M-H]-	C4H8O4	HMDB0000337
Pyrrolidine	310.09	327.97	496.48	401.37	233.01	232.11	279.42	271.84	299.69	275.09	289.35	249.07	0.01	1.64	0.71	72	[M+H]+	C4H9N	HMDB0031641
Arsenobetaine	6.58	5.12	6.24	6.84	11.93	19.88	18.40	23.65	14.12	4.84	13.93	6.88	0.01	1.71	0.71	177	[M-H]-	C5H11AsO2	HMDB0033206

Continued from the previous Table.

Name	NC1	NC2	NC3	NC4	DIO1	DIO2	DIO3	DIO4	ZBO1	ZBO2	ZBO3	ZBO4	p	vip	RT [min]	m/z	Reference Ion	Formula	HMDB
DL-Norvaline	930.84	930.32	1512.53	1237.39	741.79	722.45	855.53	825.43	918.10	865.65	897.82	743.22	0.02	1.54	0.71	118	[M+NH4]+	C5H11NO2	HMDB0251527
2,3-Dihydroxybutanoic acid	14.24	14.49	14.01	12.81	37.58	39.19	38.77	49.78	26.35	21.92	29.78	40.79	0.00	1.83	0.93	119	[M-H]-	C4H8O4	HMDB0245394
L-2-Hydroxyglutaric acid	13.29	14.60	11.68	8.86	24.62	21.21	22.41	30.06	13.28	17.45	18.85	26.70	0.00	1.63	0.93	147	[M-H]-	C5H8O5	HMDB0000694
4-hydroxy-3-nitrophenylacetate	19.52	18.31	9.77	12.19	8.19	9.02	9.07	8.19	10.91	12.16	13.02	9.67	0.02	1.67	0.93	198	[M+H]+	C8H7NO5	HMDB0062403
(R)-3-hydroxybutyrylcarnitine	47.15	36.83	29.32	40.87	58.08	56.46	74.82	96.56	38.89	47.31	60.13	68.87	0.02	1.67	0.95	248	[M+CH3OH+H]+	C11H21NO5	HMDB0062735
butyrolactone	8.96	9.95	16.16	11.91	13.21	17.28	12.15	20.99	7.23	9.84	10.02	10.78	0.04	1.68	0.99	87	[M+H]+	C4H6O2	HMDB0000549
Citramalic acid	10.49	10.30	13.31	4.76	22.12	26.35	13.82	26.35	8.81	14.61	8.66	20.03	0.04	1.52	1.06	147	[M-H]-	C5H8O5	HMDB0000426

Continued from the previous Table.

Name	NC1	NC2	NC3	NC4	DIO1	DIO2	DIO3	DIO4	ZBO1	ZBO2	ZBO3	ZBO4	p	vip	RT [min]	m/z	Reference Ion	Formula	HMDB
3-Hydroxybutyric acid	393.39	376.35	88.41	324.87	779.03	856.17	590.12	779.51	405.91	345.84	295.03	609.24	0.03	1.53	1.10	103	[M-H]-	C4H8O3	HMDB0000011
gamma-Aminobutyric acid	14.89	17.79	7.29	16.85	23.25	33.55	27.29	27.63	14.76	18.67	12.84	18.83	0.01	1.81	1.10	87	[2M+Na]+	C4H9NO2	HMDB0000112
N2,N5-Diacetylornithine	3.48	3.46	2.58	3.73	0.68	0.84	0.46	0.65	0.65	1.19	0.78	0.49	0.00	1.86	1.12	217	[M+H]+	C9H16N2O4	HMDB0240345
S-(2-carboxypropyl)-Cysteamine	6.14	5.82	2.61	2.62	8.25	8.07	9.14	10.48	6.59	6.12	8.13	9.70	0.01	1.61	1.15	164	[M+NH4]+	C6H13NO2S	HMDB0002169
Coumarin	3.85	3.22	1.60	1.30	5.30	4.48	6.10	6.65	4.14	3.64	5.40	6.21	0.01	1.59	1.15	147	[M+H]+	C9H6O2	HMDB0001218
1-Aminocyclohexanecarboxylic acid	0.71	1.07	0.99	1.50	7.60	7.18	6.96	8.32	6.72	6.79	6.36	7.58	0.00	1.92	1.24	144	[M+H]+	C7H13NO2	HMDB0243822
alpha-Hydroxyisobutyric acid	120.61	123.03	91.54	134.38	226.12	244.12	212.48	246.39	210.36	165.75	160.10	200.42	0.00	1.80	1.25	103	[M-H]-	C4H8O3	HMDB0000729

Continued from the previous Table.

Name	NC1	NC2	NC3	NC4	DIO1	DIO2	DIO3	DIO4	ZBO1	ZBO2	ZBO3	ZBO4	p	vip	RT [min]	m/z	Reference Ion	Formula	HMDB
Asp-Ile	13.39	12.80	14.12	11.91	6.98	5.66	6.97	6.56	6.94	9.18	7.22	6.34	0.00	1.78	1.50	245	[M-H]-	C10H18N2O5	HMDB0028756
Glutamylvaline	47.68	48.79	52.53	44.43	24.99	19.81	24.95	24.17	26.63	34.41	27.32	25.32	0.00	1.95	1.50	247	[M+H]+	C10H18N2O5	HMDB0028832
2',3'-Dideoxyinosine	11.74	11.64	8.05	5.11	16.25	20.32	11.56	18.47	8.44	7.60	12.48	11.54	0.04	1.57	1.58	235	[M-H]-	C10H12N4O3	HMDB0015037
2-Hydroxy-2-methylbutyric acid	7.75	7.62	7.70	8.33	6.83	5.73	6.30	5.86	7.84	6.88	8.06	6.50	0.01	1.76	1.72	117	[M-H]-	C5H10O3	HMDB0001987
O-3-methylglutarylcarnitine	8.47	8.29	5.19	4.54	15.93	13.66	16.10	18.13	8.76	16.06	13.50	14.85	0.00	1.77	1.86	290	[M+H]+	C13H23NO6	HMDB0000552
gamma-Glutamyltyrosine	32.55	33.04	32.01	25.33	18.78	10.82	16.72	14.40	11.47	18.71	16.45	17.42	0.00	1.73	1.93	311	[M+H]+	C14H18N2O6	HMDB0011741

Pantothenate	39.37	38.94	57.88	42.45	100.80	64.96	72.10	116.97	54.91	93.22	76.22	73.49	0.01	1.67	2.17	220	[M+H]+	C9H17NO5	HMDB0000210
2-Hydroxy-3-methylbutyric acid	142.59	143.60	189.92	155.30	213.52	251.48	209.49	231.96	196.91	134.58	175.73	202.43	0.01	1.66	2.93	117	[M-H]-	C5H10O3	HMDB0000407

Continued from the previous Table.

Name	NC1	NC2	NC3	NC4	DIO1	DIO2	DIO3	DIO4	ZBO1	ZBO2	ZBO3	ZBO4	p	vip	RT [min]	m/z	Reference Ion	Formula	HMDB
2,5-Dihydroxybenzenesulfonic Acid	7.03	7.13	7.46	17.23	1.20	1.48	4.63	0.82	2.97	3.44	2.23	4.38	0.00	1.65	3.09	189	[M-H]-	C6H6O5S	HMDB0245498
gamma-Glutamylleucine	97.72	95.12	122.03	98.14	58.09	42.97	53.69	53.04	55.30	61.24	53.25	55.89	0.00	1.89	3.12	261	[M+H]+	C11H20N2O5	HMDB0011171
glutaral	2.50	2.53	2.39	1.94	6.28	5.16	2.35	3.37	1.32	1.87	1.65	1.40	0.00	1.98	3.17	83	[M+H-H2O]+	C5H8O2	HMDB0029599
gamma-Glutamylphenylalanine	18.99	19.44	20.52	18.53	13.15	9.39	12.03	11.74	10.82	13.68	11.61	11.81	0.00	1.74	3.25	293	[M-H]-	C14H18N2O5	HMDB0000594

Glutamylphenylalanine	50.90	50.98	52.83	49.63	35.24	23.56	30.38	30.56	27.70	35.98	31.41	31.23	0.00	1.82	3.25	295	[M+H]+	C14H18N2O5	HMDB0029156
alpha-oxycodol	6.48	7.22	5.84	7.55	11.10	13.01	10.04	9.91	8.95	6.75	10.04	9.71	0.00	1.83	3.28	318	[M+H]+	C18H23NO4	HMDB0061077

Continued from the previous Table.

Name	NC1	NC2	NC3	NC4	DIO1	DIO2	DIO3	DIO4	ZBO1	ZBO2	ZBO3	ZBO4	p	vip	RT [min]	m/z	Reference Ion	Formula	HMDB
Isovalerylcarnitine	48.35	45.93	50.15	45.75	31.51	24.57	26.69	35.45	30.07	36.66	36.07	33.88	0.00	1.87	3.35	246	[M+H]+	C12H23NO4	HMDB0000688
Glu-Trp	16.22	16.20	15.19	15.75	12.50	7.46	11.46	10.81	7.72	11.73	9.61	11.01	0.01	1.56	3.39	334	[M+H]+	C16H19N3O5	HMDB0028830
Hippuric acid	3.04	3.14	4.19	3.97	1.67	1.30	1.37	1.78	1.06	1.59	1.27	1.14	0.00	1.79	3.45	180	[M+H]+	C9H9NO3	HMDB0000714
4-oxo-4-(3-pyridyl)butanoic acid	11.28	11.43	20.11	16.24	4.48	2.94	4.67	5.93	4.06	6.03	3.90	4.60	0.00	1.71	3.45	178	[M-H]-	C9H9NO3	HMDB0000992
trequinsin	13.14	12.77	10.65	8.74	7.39	6.98	8.24	6.18	11.96	8.23	13.88	8.43	0.02	1.64	3.49	404	[M-H]-	C24H27N3O3	HMDB0259121

2-Hydroxy-5-octenoylcarnitine	10.69	10.92	6.99	6.35	12.50	12.30	19.24	17.11	12.06	11.45	11.91	17.22	0.02	1.56	3.53	302	[M+H]+	C15H27NO5	HMDB0241703
2-Amino-5,6-dichloro-3,4,-dihydroquinazoline	1.15	1.02	1.20	1.27	2.63	2.39	2.19	2.87	2.17	1.39	2.00	1.97	0.00	1.83	3.59	214	[M-H]-	C8H7Cl2N3	HMDB0060712
3-Methoxyphenol sulfate	1.55	1.43	1.98	1.81	2.90	1.74	4.56	2.36	5.16	4.44	3.74	4.69	0.00	1.69	3.66	203	[M-H]-	C7H8O5S	HMDB0304914

Continued from the previous Table.

Name	NC1	NC2	NC3	NC4	DIO1	DIO2	DIO3	DIO4	ZBO1	ZBO2	ZBO3	ZBO4	p	vip	RT [min]	m/z	Reference Ion	Formula	HMDB
PGP(i-14:0/5-iso PGF2VI)	1.09	1.04	1.78	1.32	1.55	2.32	1.72	4.15	0.64	1.40	1.23	1.37	0.05	1.60	3.68	843	[M-H]-	C38H70O16P2	HMDB0275037
1-Methoxypyrene	2.77	2.82	2.76	2.70	1.55	1.00	1.51	1.34	1.02	2.00	1.65	1.25	0.00	1.64	3.72	231	[M-H]-	C17H12O	HMDB0060324
2-Hydroxydecanedioic acid	6.46	6.67	4.76	3.52	2.99	3.55	4.01	2.84	4.58	5.67	6.19	4.58	0.02	1.68	3.74	217	[M-H]-	C10H18O5	HMDB0000424
Pseudoecgonine	0.37	0.44	0.60	0.41	2.02	1.78	2.31	2.56	1.31	1.74	1.47	2.19	0.00	1.82	3.76	184	[M-H]-	C9H15NO3	HMDB0006348

Hexanoylglycine	156.13	159.09	63.93	117.38	54.88	70.85	54.05	60.00	39.80	66.28	48.74	53.54	0.01	1.53	3.81	172	[M-H]-	C8H15NO3	HMDB0000701
Glycine	3.55	3.68	1.57	2.78	1.37	1.82	1.51	1.51	1.06	1.61	1.31	1.32	0.01	1.52	3.81	74	[M-H]-	C2H5NO2	HMDB0000123
2,3-Epoxysesamone	0.67	0.55	0.32	0.68	1.00	3.73	2.35	1.29	1.03	2.24	1.73	0.90	0.01	1.50	3.85	273	[M-H]-	C15H14O5	HMDB0033333

Continued from the previous Table.

Name	NC1	NC2	NC3	NC4	DIO1	DIO2	DIO3	DIO4	ZBO1	ZBO2	ZBO3	ZBO4	p	vip	RT [min]	m/z	Reference Ion	Formula	HMDB
2,3-Methylene suberic acid	5.84	5.51	3.30	4.15	8.01	12.91	12.61	13.96	7.04	12.67	7.07	12.93	0.00	1.60	3.86	197	[M-H]-	C10H14O4	HMDB0059779
Indolelacyic acid	43.15	43.12	38.30	42.26	58.43	65.34	74.79	70.92	53.88	51.14	65.58	71.23	0.00	1.70	3.86	204	[M-H]-	C11H11NO3	HMDB0000671

Name	NC1	NC2	NC3	NC4	DIO1	DIO2	DIO3	DIO4	ZBO1	ZBO2	ZBO3	ZBO4	p	vip	RT [min]	m/z	Reference Ion	Formula	HMDB
N-(Cinnamoyl) Glycine	8.78	8.52	7.07	16.85	0.96	1.75	1.86	1.77	1.91	2.30	1.43	1.99	0.00	1.78	3.95	204	[M-H]-	C11H11NO3	HMDB0011621
1-Octen-3-one	3.45	3.31	4.31	0.93	0.56	0.86	0.64	0.94	0.64	0.65	0.87	0.52	0.00	1.57	3.95	125	[M-H]-	C8H14O	HMDB0031309
Lobenzarit	3.24	3.45	5.05	1.02	0.66	0.79	0.59	0.71	0.58	0.72	0.89	0.58	0.03	1.62	3.95	290	[M-H]-	C14H10ClNO4	HMDB0254140
5-(3',4'-Dihydroxyphenyl)-gamma-valerolactone 4'-sulfate	1.80	1.75	2.18	0.62	0.31	0.41	0.37	0.41	0.23	0.31	0.52	0.29	0.00	1.64	3.95	287	[M-H]-	C11H12O7S	HMDB0240473

Continued from the previous Table.

Name	NC1	NC2	NC3	NC4	DIO1	DIO2	DIO3	DIO4	ZBO1	ZBO2	ZBO3	ZBO4	p	vip	RT [min]	m/z	Reference Ion	Formula	HMDB
2'-Hydroxy-4',6'-dimethoxy-3'-methylacetophenone	2.73	2.37	3.21	0.77	0.38	0.63	0.36	0.77	0.44	0.51	0.68	0.41	0.00	1.59	3.95	209	[M-H]-	C11H14O4	HMDB0034047

Azelaic acid	167.21	169.46	237.97	52.58	37.18	45.14	34.20	46.86	35.54	37.41	50.34	33.61	0.04	1.58	3.95	187	[M-H]-	C9H16O4	HMDB0000784
4-Ethylphenylsulfate	30.46	29.91	24.27	15.85	2.13	8.33	1.36	2.27	4.00	2.43	2.34	1.35	0.00	1.69	4.04	201	[M-H]-	C8H10O4S	HMDB0062551
7,7'-Dihydroxy-6,8'-bicoumarin	35.64	34.82	31.44	43.87	0.71	2.79	1.15	1.48	1.72	1.06	1.11	0.95	0.00	1.81	4.04	321	[M-H]-	C18H10O6	HMDB0033559
Saxitoxin	11.19	10.92	10.58	10.68	10.29	10.71	10.69	10.46	11.13	11.32	10.99	11.25	0.01	1.88	4.09	300	[M+H]+	C10H17N7O4	HMDB0029368
Aminofurantoin	4.25	6.98	5.04	4.80	0.39	0.43	0.35	0.52	0.53	0.87	0.36	0.37	0.00	1.82	4.24	207	[M-H]-	C8H8N4O3	HMDB0060589

Continued from the previous Table.

Name	NC1	NC2	NC3	NC4	DIO1	DIO2	DIO3	DIO4	ZBO1	ZBO2	ZBO3	ZBO4	p	vip	RT [min]	m/z	Reference Ion	Formula	HMDB
3-(Cyclohexylamino)-2-hydroxy-1-propanesulfonic acid	9.64	9.83	8.13	7.92	7.23	8.06	7.87	8.41	6.53	7.56	7.19	6.31	0.01	1.56	4.20	236	[M-H]-	C9H19NO4S	HMDB0247339

Cacalol	2.91	3.04	2.52	1.40	3.99	8.75	6.26	7.21	3.17	4.73	4.49	6.45	0.01	1.71	4.20	231	[M+H]+	C15H18O2	HMDB0249525
3-Decenoylcarnitine	21.22	19.97	26.59	22.32	13.77	19.51	11.97	21.25	9.54	11.03	10.18	11.85	0.00	1.68	4.36	314	[M+H]+	C17H31NO4	HMDB0241067
11beta,20-Dihydroxy-3-oxopregn-4-en-21-oic acid	1.70	1.38	2.22	3.09	5.81	3.12	5.96	9.70	3.05	2.25	2.32	5.66	0.02	1.57	4.37	361	[M-H]-	C21H30O5	HMDB0011651
Cortisol	0.89	0.66	1.07	1.71	3.03	1.47	3.01	5.21	1.71	1.17	1.25	2.97	0.03	1.62	4.37	363	[M+Na]+	C21H30O5	HMDB0000063
Adibendan	3.12	3.01	2.85	2.40	8.08	9.64	7.69	8.88	4.84	5.85	5.57	7.40	0.00	1.88	4.37	277	[M-H]-	C16H14N4O	HMDB0248011
Dillanol	0.19	0.09	0.30	0.06	10.22	9.25	10.89	9.96	9.98	8.48	9.79	11.15	0.00	1.82	4.43	307	[M-H]-	C18H12O5	HMDB0302237

Continued from the previous Table.

Name	NC1	NC2	NC3	NC4	DIO1	DIO2	DIO3	DIO4	ZBO1	ZBO2	ZBO3	ZBO4	p	vip	RT [min]	m/z	Reference Ion	Formula	HMDB
Capryloyl Glycine	22.46	23.46	9.61	15.40	3.00	7.18	5.94	9.12	3.88	6.70	4.96	6.53	0.00	1.54	4.43	200	[M-H]-	C10H19NO3	HMDB0000832

3-hydroxydodecanoyl carnitine	4.68	4.32	6.02	6.20	9.02	10.77	9.72	12.55	7.09	7.22	11.91	9.53	0.00	1.77	4.47	360	[M+H]+	C19H37NO5	HMDB0061638
4-(7-Hydroxy-6,7-dihydro-5H-cyclopenta[d]pyrimidin-4-ylamino)benzonitrile	5.30	6.53	3.47	3.69	8.57	17.27	8.09	15.10	9.86	5.27	3.94	3.86	0.02	1.70	4.49	251	[M-H]-	C14H12N4O	HMDB0244317
DECANOYL CARNITINE	17.31	17.19	24.00	17.47	23.21	33.65	21.04	46.98	16.37	17.56	21.95	22.05	0.05	1.63	4.50	316	[M+H]+	C17H33NO4	HMDB0000651
11-Dehydrocorticosterone	4.67	4.62	3.95	6.37	3.31	1.66	2.76	2.75	3.30	2.63	3.16	2.80	0.01	1.68	4.50	345	[M+ACN+H]2+	C21H28O4	HMDB0004029
3-Aminosalicylic acid	1.81	2.06	1.56	1.20	1.01	1.15	0.96	1.18	1.09	1.20	0.96	0.97	0.01	1.53	4.55	152	[M-H]-	C7H7NO3	HMDB0001972

Continued from the previous Table.

Name	NC1	NC2	NC3	NC4	DIO1	DIO2	DIO3	DIO4	ZBO1	ZBO2	ZBO3	ZBO4	p	vip	RT [min]	m/z	Reference Ion	Formula	HMDB
Mequitazine	1.14	0.07	0.10	0.07	13.46	17.06	12.88	13.96	12.18	13.63	11.07	12.42	0.00	1.76	4.58	321	[M-H]-	C20H22N2S	HMDB0015204
N-Octanoyl-L-Homoserine Lactone	7.22	7.21	3.95	5.25	2.80	1.58	4.14	1.95	4.07	3.90	5.54	3.87	0.01	1.68	4.59	226	[M-H]-	C12H21NO3	HMDB0255213
Dihydrodaidzein	5.95	6.52	6.31	5.55	5.17	5.30	5.30	5.61	5.15	5.71	5.17	5.05	0.01	1.52	4.60	163	[M+H]+	C15H12O4	HMDB0005760
Dodecanedioic acid	2.35	2.51	4.13	2.68	5.33	8.41	4.62	7.47	4.82	5.73	5.15	4.46	0.00	1.66	4.61	229	[M-H]-	C12H22O4	HMDB0000623
16alpha-Hydroxyprednisolone	5.10	5.50	11.46	0.75	0.14	0.34	0.54	0.54	0.59	0.46	0.21	0.20	0.00	1.57	4.64	375	[M-H]-	C21H28O6	HMDB0244573
Matricarin	3.62	3.09	3.04	2.98	8.34	8.50	6.27	7.95	4.01	3.99	4.99	6.62	0.00	1.89	4.64	303	[M-H]-	C17H20O5	HMDB0035790
5-Dodecenoylcarnitine	10.95	10.84	9.01	9.35	12.48	15.17	11.69	22.77	7.69	9.84	10.14	12.17	0.03	1.71	4.66	342	[M+H]+	C19H35NO4	HMDB0241212
Pretazettine	4.18	4.06	1.44	1.31	0.31	0.22	0.39	0.28	0.28	0.35	0.52	0.21	0.00	1.72	4.67	330	[M-H]-	C18H21NO5	HMDB0302721

Continued from the previous Table.

Name	NC1	NC2	NC3	NC4	DIO1	DIO2	DIO3	DIO4	ZBO1	ZBO2	ZBO3	ZBO4	p	vip	RT [min]	m/z	Reference Ion	Formula	HMDB
10-(Phosphonooxy)decyl methacrylate	13.37	11.53	16.11	12.40	33.97	41.44	40.75	46.48	34.32	36.68	44.90	38.97	0.00	1.81	4.70	321	[M-H]-	C14H27O6P	HMDB0247525
Albuterol	9.60	9.45	2.59	5.61	1.12	0.60	2.45	0.42	3.19	2.17	4.62	2.46	0.01	1.74	4.71	238	[M-H]-	C13H21NO3	HMDB0001937
3,5-Pyridinedicarboxylic acid, 1,4-dihydro-2,4,6-trimethyl-, diethyl ester	2.86	2.87	3.58	2.18	0.16	0.18	0.11	0.18	0.03	0.09	0.03	0.02	0.00	1.84	4.72	266	[M-H]-	C14H21NO4	HMDB0247156
(9E)-Dodecenoylcarnitine	1.74	1.41	2.48	2.24	2.99	4.13	3.86	4.82	2.37	2.24	4.16	3.57	0.01	1.68	4.74	342	[M+H]+	C19H35NO4	HMDB0241209
Sphingosine 1-phosphate (d16:1-P)	3.71	3.18	4.16	3.48	6.61	11.64	7.96	9.57	8.71	7.89	9.79	8.14	0.00	1.75	4.75	350	[M-H]-	C16H34NO5P	HMDB0060061
2-Carboxy-4-dodecanolide	1.40	1.30	1.72	1.88	4.11	4.59	3.67	4.76	4.01	4.04	2.10	3.73	0.00	1.74	4.78	241	[M-H]-	C13H22O4	HMDB0030987

Continued from the previous Table.

Name	NC1	NC2	NC3	NC4	DIO1	DIO2	DIO3	DIO4	ZBO1	ZBO2	ZBO3	ZBO4	p	vip	RT [min]	m/z	Reference Ion	Formula	HMDB
(-)-O-Desmethyl Tramadol	9.54	6.10	9.75	7.37	3.48	4.72	3.23	4.45	4.15	8.81	8.20	3.61	0.03	1.61	4.80	250	[M+H]+	C15H23NO2	HMDB0060997
Deoxyspergualin	3.99	2.18	3.25	4.40	5.76	9.52	7.63	10.70	6.97	5.67	9.61	7.44	0.00	1.71	4.80	388	[M+H]+	C17H37N7O3	HMDB0243514
Inolin	23.28	24.17	10.35	9.90	5.07	7.15	6.44	6.73	4.79	5.23	6.33	6.15	0.00	1.59	4.83	344	[M-H]-	C19H23NO5	HMDB0253488
Tridecanedioic acid	5.33	5.97	8.64	6.14	24.85	22.81	19.07	28.15	17.65	17.08	18.68	19.21	0.00	1.83	4.83	243	[M-H]-	C13H24O4	HMDB0002327
5(S),15(S)-DiHETE	10.06	10.69	13.71	7.12	0.95	2.37	2.65	1.26	1.92	2.74	3.69	2.64	0.00	1.77	4.84	371	[M-H]-	C20H32O4	HMDB0010216
3-Hydroxypalmitoleoylcarnitine	4.82	3.70	4.22	4.94	10.97	14.77	13.09	17.77	9.63	8.73	13.88	13.96	0.00	1.89	4.94	414	[M+H]+	C23H43NO5	HMDB0240770
Hexadeca-7,10,13-	9.11	8.68	11.99	10.53	12.62	17.11	12.56	18.75	9.01	10.90	11.22	13.03	0.02	1.76	4.97	394	[M+H]+	C23H39NO4	HMDB0241494

trienoylcarnitine																		

Continued from the previous Table.

Name	NC1	NC2	NC3	NC4	DIO1	DIO2	DIO3	DIO4	ZBO1	ZBO2	ZBO3	ZBO4	p	vip	RT [min]	m/z	Reference Ion	Formula	HMDB
cis-5-Tetradecenoylcarnitine	84.74	81.21	79.94	69.58	121.91	170.50	129.21	202.85	102.71	101.59	123.94	137.45	0.00	1.88	4.98	370	[M+H]+	C21H39NO4	HMDB0002014
5-Nonyltetrahydro-2-oxo-3-furancarboxylic acid	1.80	2.03	2.26	1.86	7.46	5.70	6.27	6.88	4.65	5.18	5.39	6.36	0.00	1.84	4.98	255	[M-H]-	C14H24O4	HMDB0030993
(6E,8R,10Z)-8-hydroxy-3-oxohexadecadienoic acid	1.24	1.25	1.90	1.52	7.80	7.54	6.18	7.62	4.92	4.92	4.59	6.07	0.00	1.86	5.00	281	[M-H]-	C16H26O4	HMDB0062365
Tetradecanedioic acid	3.91	4.12	9.93	6.17	13.80	16.23	10.85	19.16	8.25	8.30	8.20	11.37	0.00	1.69	5.01	257	[M-H]-	C14H26O4	HMDB0000872
3-Oxododecanoic acid	18.86	19.54	23.69	16.79	12.70	25.21	10.88	23.77	9.12	7.81	12.86	10.54	0.02	1.58	5.07	213	[M-H]-	C12H22O3	HMDB0010727

(2S)-2-Hydroxyhexadecanoylcarnitine	4.76	3.95	6.15	5.48	8.52	13.03	10.87	14.64	8.50	7.12	12.83	12.60	0.00	1.73	5.11	416	[M+H]+	C23H45NO5	HMDB0241459

Continued from the previous Table.

Name	NC1	NC2	NC3	NC4	DIO1	DIO2	DIO3	DIO4	ZBO1	ZBO2	ZBO3	ZBO4	p	vip	RT [min]	m/z	Reference Ion	Formula	HMDB
(Z)-7-[(1R,2R,3R,5S)-2-(1,2-Dihydroxy-3-oxooctyl)-3,5-dihydroxycyclopentyl]hept-5-enoic acid	15.84	15.79	15.96	11.59	1.87	5.44	5.68	2.22	5.06	6.97	9.47	5.71	0.00	1.74	5.12	385	[M-H]-	C20H34O7	HMDB0244520
Myristoyl-carnitine	69.08	65.13	67.27	64.95	84.61	130.88	90.18	138.75	78.53	66.92	101.20	100.12	0.03	1.69	5.14	372	[M+H]+	C21H41NO4	HMDB0254979
Bardoxolone	9.87	9.19	11.46	10.55	4.33	6.23	4.60	6.06	4.86	4.99	4.30	5.48	0.00	1.80	5.10	492	[M+H]+	C31H41NO4	HMDB0248871

(5Z,8Z,11Z,14Z,17Z)-Icosa-5,8,11,14,17-pentaenoylcarnitine	5.47	4.93	8.43	13.78	2.40	3.74	2.23	3.96	2.34	2.20	3.27	2.67	0.00	1.61	5.15	446	[M+H]+	C27H43NO4	HMDB0241609
Tetranorprostanedioic acid	3.53	4.31	8.68	5.75	13.25	14.85	9.75	15.15	8.14	9.38	8.65	9.52	0.00	1.70	5.23	283	[M-H]-	C16H28O4	HMDB0258926

Continued from the previous Table.

Name	NC1	NC2	NC3	NC4	DIO1	DIO2	DIO3	DIO4	ZBO1	ZBO2	ZBO3	ZBO4	p	vip	RT [min]	m/z	Reference Ion	Formula	HMDB
LysoPC(18:3(6Z,9Z,12Z)/0:0)	9.18	8.46	15.52	10.75	4.27	5.16	4.28	5.06	4.43	3.28	3.47	3.60	0.00	1.77	5.18	518	[M+H]+	C26H48NO7P	HMDB0010387
3-Hydroxy-11Z-octadecenoylcarnitine	6.72	6.60	8.01	6.68	16.86	22.89	18.61	24.40	15.07	13.85	22.86	21.82	0.00	1.87	5.21	442	[M+H]+	C25H47NO5	HMDB0013339
LysoPE(0:0/18:3(9Z,12Z,15Z))	5.70	5.90	15.68	2.95	1.66	5.11	1.35	3.01	0.34	0.87	0.80	1.40	0.00	1.59	5.22	476	[M+H]+	C23H42NO7P	HMDB0011479

LysoPE(18:3(9Z,12Z,15Z)/0:0)	5.22	5.26	13.85	3.92	2.37	4.90	2.22	3.19	1.85	1.45	1.46	2.25	0.00	1.60	5.22	474	[M-H]-	C23H42NO7P	HMDB0011509
7-Hexadecenoylcarnitine	79.70	79.20	67.75	79.94	105.09	148.51	110.57	162.33	90.06	81.83	107.16	126.15	0.01	1.78	5.22	398	[M+H]+	C23H43NO4	HMDB0241472
LysoPE(20:5(5Z,8Z,11Z,14Z,17Z)/0:0)	19.17	18.14	39.73	22.77	5.93	12.65	5.20	8.60	5.23	3.80	4.59	5.86	0.00	1.72	5.23	498	[M-H]-	C25H42NO7P	HMDB0011519

Continued from the previous Table.

Name	NC1	NC2	NC3	NC4	DIO1	DIO2	DIO3	DIO4	ZBO1	ZBO2	ZBO3	ZBO4	p	vip	RT [min]	m/z	Reference Ion	Formula	HMDB
LysoPE(0:0/20:5(5Z,8Z,11Z,14Z,17Z))	19.99	20.86	45.42	24.72	6.74	14.46	5.55	9.39	6.29	3.94	5.32	6.61	0.00	1.69	5.24	500	[M+H]+	C25H42NO7P	HMDB0011489
LysoPC(20:5(5Z,8Z,11Z,14Z,17Z)/0:0)	149.94	140.29	238.96	285.42	80.10	81.99	65.96	89.15	70.32	69.94	76.75	65.79	0.01	1.75	5.25	542	[M+H]+	C28H48NO7P	HMDB0010397
1-palmitoyl-2-	8.53	2.35	8.98	6.36	2.57	2.91	1.72	5.31	8.08	7.69	6.36	17.67	0.03	1.70	5.26	827	[M-H]-	C44H80NO8P	HMDB0007982

arachidonoyl-sn-glycero-3-phosphocholine																		
Tyrosine methylester	61.87	63.77	62.08	59.22	59.53	58.87	58.97	57.54	60.78	65.71	64.64	63.83	0.01	1.88	5.27	194	[M-H]-	C10H13NO3	HMDB0029217
3-Hydroxydodecanoic acid	10.71	11.07	9.36	9.05	21.21	32.96	13.81	30.57	13.98	8.52	21.20	13.43	0.01	1.63	5.30	215	[M-H]-	C12H24O3	HMDB0000387
Linoleyl carnitine	202.84	212.70	231.33	205.24	126.86	200.79	148.29	204.60	122.47	107.75	134.07	157.33	0.01	1.53	5.30	424	[M+H]+	C25H45NO4	HMDB0240780
LysoPE(18:2(9Z,12Z)/0:0)	45.56	45.95	89.30	34.84	17.95	45.09	19.86	26.84	21.57	15.27	15.95	21.63	0.01	1.53	5.39	476	[M-H]-	C23H44NO7P	HMDB0011507

Continued from the previous Table.

Name	NC1	NC2	NC3	NC4	DIO1	DIO2	DIO3	DIO4	ZBO1	ZBO2	ZBO3	ZBO4	p	vip	RT [min]	m/z	Reference Ion	Formula	HMDB
Palmitoyl-carnitine	331.83	342.36	360.04	354.11	420.31	619.83	402.85	551.57	363.17	341.08	368.96	442.76	0.02	1.78	5.41	400	[M+H]+	C23H45NO4	HMDB0240783
Oleoyl-carnitine	212.01	218.62	196.89	201.27	340.51	467.13	401.46	459.38	295.58	294.98	334.15	412.74	0.00	1.94	5.47	426	[M+H]+	C25H47NO4	HMDB0005065
LysoPC(18:2(9Z,12Z)/0:0)	11121.44	10906.43	15971.59	13354.07	7709.05	10710.65	8141.82	9861.54	9942.47	8046.67	8353.45	8190.47	0.01	1.52	5.49	520	[M+H]+	C26H50NO7P	HMDB0010386

Name	NC1	NC2	NC3	NC4	DIO1	DIO2	DIO3	DIO4	ZBO1	ZBO2	ZBO3	ZBO4	p	vip	RT [min]	m/z	Reference Ion	Formula	HMDB
3-Oxotetradecanoic acid	8.20	8.30	7.30	7.62	14.38	19.85	10.18	22.69	8.01	6.03	9.69	10.21	0.01	1.84	5.51	241	[M-H]-	C14H26O3	HMDB0010730
Diacetoxyscirpenol	4.24	2.82	3.43	3.93	1.63	1.72	3.69	4.30	5.48	4.34	9.56	6.06	0.03	1.71	5.51	365	[M-H]-	C19H26O7	HMDB0035104
1-hexadecanoyl-2-(4Z,7Z,10Z,13Z,16Z,19Z-docosahexaenoyl)-sn-glycero-3-phosphocholine	10.49	7.44	17.37	10.37	7.36	19.06	32.30	19.88	34.71	51.82	114.28	39.17	0.01	1.61	5.53	807	[M+ACN+H]+	C46H80NO8P	HMDB0007991

Continued from the previous Table.

Name	NC1	NC2	NC3	NC4	DIO1	DIO2	DIO3	DIO4	ZBO1	ZBO2	ZBO3	ZBO4	p	vip	RT [min]	m/z	Reference Ion	Formula	HMDB
5-[[5-[[5-(Pyrrol-2-	1.76	1.85	1.66	1.40	4.13	6.75	4.15	7.60	2.41	2.50	2.99	4.13	0.00	1.86	5.59	341	[M-H]-	C20H14N4O2	HMDB0260427

ylidenemethyl)pyrrol-2-ylidene]methyl]-1H-pyrrol-2-yl]methylidene]pyrrole-2-carboxylic acid																		
12-HEPE	51.56	55.52	99.11	103.62	31.71	49.82	17.95	32.60	21.82	20.46	26.63	17.52	0.00	1.63	5.60	317	[M-H]1-	C20H30O3	HMDB0010202
Brassinolide	2.98	2.82	3.77	3.58	3.93	4.91	4.80	4.51	4.83	5.39	4.41	4.26	0.00	1.57	5.63	479	[M-H]-	C28H48O6	HMDB0034081
cholesteryl sulfate	1.20	3.38	3.10	2.74	0.96	2.74	2.18	0.54	4.31	2.52	6.64	9.39	0.04	1.67	5.63	465	[M-H]-	C27H46O4S	HMDB0000653
LysoPE(20:3(11Z,14Z,17Z)/0:0)	12.47	11.82	27.51	12.66	6.00	15.86	5.61	8.59	5.95	4.53	4.23	5.62	0.01	1.56	5.64	502	[M-H]-	C25H46NO7P	HMDB0011514

Continued from the previous Table.

Name	NC1	NC2	NC3	NC4	DIO1	DIO2	DIO3	DIO4	ZBO1	ZBO2	ZBO3	ZBO4	p	vip	RT [min]	m/z	Reference Ion	Formula	HMDB

Stearoyl-L-carnitine	9.22	8.84	16.72	12.73	22.36	44.66	26.64	39.34	23.53	21.07	33.34	35.73	0.00	1.73	5.64	428	[2M+Na]+	C25H49NO4	HMDB0000848
(+/-)12(13)-DiHOME	10.06	9.25	9.46	10.78	3.36	5.28	3.30	4.34	4.96	3.51	5.41	2.08	0.00	1.61	5.69	295	[M-H]-	C18H34O4	HMDB0004705
Omega-Hydroxydodecanoate	12.08	12.30	10.74	8.85	4.35	2.16	6.35	4.15	1.63	2.20	2.32	4.31	0.00	1.66	5.69	239	[M+H]+	C12H24O3	HMDB0002059
LysoPC(22:5(7Z,10Z,13Z,16Z,19Z)/0:0)	14.70	12.36	26.01	29.42	80.09	119.72	101.70	97.37	143.14	105.92	97.03	112.27	0.00	1.82	5.71	570	[M+H]+	C30H52NO7P	HMDB0010403
(R)-3-Hydroxy myristic acid	5.75	5.54	5.30	4.78	9.02	15.59	6.23	16.77	5.85	4.21	7.86	6.98	0.02	1.64	5.79	243	[M-H]-	C14H28O3	HMDB0010731
LysoPC(22:4(7Z,10Z,13Z,16Z)/0:0)	12.30	11.76	23.46	23.73	34.06	45.02	30.57	40.16	43.22	37.68	34.19	34.05	0.00	1.66	5.85	572	[M+H]+	C30H54NO7P	HMDB0010401
(+/-)9-HpODE	3.07	3.33	4.38	2.85	6.68	11.25	4.46	8.93	4.23	3.84	3.30	4.16	0.00	1.82	5.91	293	[M-H]-	C18H32O4	HMDB0242602
Gomisin J	0.04	0.05	0.05	0.03	5.21	5.42	5.08	5.71	4.19	4.73	4.74	6.33	0.00	1.84	5.92	387	[M-H]-	C22H28O6	HMDB0252912

Continued from the previous Table.

Name	NC1	NC2	NC3	NC4	DIO1	DIO2	DIO3	DIO4	ZBO1	ZBO2	ZBO3	ZBO4	p	vip	RT [min]	m/z	Reference Ion	Formula	HMDB

3-Oxohexadecanoic acid	7.48	7.50	6.68	5.36	14.68	24.87	10.42	26.38	8.97	6.62	11.83	9.80	0.00	1.77	5.98	269	[M-H]-	C16H30O3	HMDB0010733
3a,20b-Pregnanediol	0.09	0.30	0.37	0.42	4.33	5.31	4.82	5.20	9.50	4.77	3.87	4.27	0.00	1.77	6.01	319	[M-H]-	C21H36O2	HMDB0002156
D8'-Merulinic acid A	3.28	1.84	2.72	2.02	11.73	12.00	10.85	12.27	10.56	11.85	11.62	12.16	0.00	1.82	6.06	389	[M-H]-	C24H38O4	HMDB0041454
2-Arachidonyl glycerol ether	0.44	0.24	0.35	0.50	4.19	5.22	5.33	5.27	5.05	5.06	4.53	5.04	0.00	1.83	6.16	409	[M-H]-	C23H40O3	HMDB0013657
6,11-Dihydroxy-3-methyl-3-(4-methyl-3-pentenyl)-3H,7H-pyrano[2,3-c]xanthen-7-one	11.03	10.87	9.22	10.92	4.46	6.14	3.37	6.76	4.73	3.29	3.78	3.36	0.00	1.71	6.47	377	[M-H]-	C23H22O5	HMDB0040350

Continued from the previous Table.

Name	NC1	NC2	NC3	NC4	DIO1	DIO2	DIO3	DIO4	ZBO1	ZBO2	ZBO3	ZBO4	p	vip	RT [min]	m/z	Reference Ion	Formula	HMDB
Furost-5-ene-3beta,22,26-triol	7.18	6.53	9.34	7.98	49.79	60.42	46.03	49.13	53.65	57.71	45.50	44.17	0.00	1.83	6.50	417	[M-H]-	C26H42O4	HMDB0303029
iso deoxycholic acid	2.43	3.23	3.71	3.40	27.50	34.68	33.28	32.48	34.22	37.60	29.24	30.61	0.00	1.84	6.28	391	[M-H]-	C24H40O4	HMDB0002536
3-Oxooctadecanoic acid	5.66	5.39	4.29	3.51	10.14	20.11	6.72	19.69	7.22	5.00	6.81	6.43	0.01	1.73	6.46	297	[M-H]-	C18H34O3	HMDB0010736
Hydroxydesmethyltetrahydropiperine sulfate	2.53	2.72	1.38	2.18	1.95	2.59	0.79	2.40	1.25	0.80	0.90	0.71	0.03	1.54	6.71	372	[M-H]-	C16H23NO7S	HMDB0304921
Linalyl caprylate	6.80	7.38	6.26	5.76	2.71	4.02	1.90	3.33	2.81	1.71	2.17	1.35	0.00	1.70	6.87	279	[M-H]-	C18H32O2	HMDB0030430

Continued from the previous Table.

Name	NC1	NC2	NC3	NC4	DIO1	DIO2	DIO3	DIO4	ZBO1	ZBO2	ZBO3	ZBO4	p	vip	RT [min]	m/z	Reference Ion	Formula	HMDB
(3-{[3-(2-Amino-2-Oxoethyl)-1-Benzyl-2-Ethyl-1h-Indol-5-Yl]oxy}propyl)phosphonic Acid	1.71	1.60	1.46	1.23	0.70	0.45	0.32	1.12	0.54	0.57	0.51	0.25	0.00	1.54	6.87	429	[M-H]-	C22H27N2O5P	HMDB0254234

Continued from the previous Table
